# Immunological and clinicopathological characteristics of *C1RL* in 2120 glioma patients

**DOI:** 10.1186/s12885-020-07436-6

**Published:** 2020-09-29

**Authors:** Junyou Wang, Luqing Tong, Gaojun Lin, Hui Wang, Liang Zhang, Xuejun Yang

**Affiliations:** 1 Department of Neurosurgery, The First People’s Hospital of Wenling, Wenling, 317500 China; 2 Department of Neurosurgery, Tianjin Medical University General Hospital, Tianjin, 300052 China; 3 Department of Neurosurgery, The First Affiliated Hospital of Medical School of Zhejiang University, Hangzhou, 310003 China; 4 Department of Neurosurgery, The Johns Hopkins University School of Medicine, Baltimore, 21287 USA

**Keywords:** Glioma, *C1RL*, Immunosuppression, Unfavourable survival, Therapeutic resistance

## Abstract

**Background:**

Glioma is a deadly and immunosuppressive brain tumour. Complement C1r subcomponent like (*C1RL*), a prognostic biomarker in several kinds of tumours, has attracted increasing attention from oncologists. However, the role of *C1RL* in glioma remains unclear.

**Methods:**

Through analysis of 2120 glioma patients from 5 public datasets, the relationships between *C1RL* expression and clinicopathological characteristics were evaluated. Furthermore, the *C1RL*-associated genes were screened, and Gene Ontology (GO) analysis was conducted to investigate biological process enrichment. In addition, tumour purity, leukocyte infiltration and overall survival were evaluated based on *C1RL* expression.

**Results:**

We found that *C1RL* expression was upregulated in glioblastoma (GBM), especially mesenchymal GBM and primary GBM. Increased *C1RL* expression accompanied the *IDH1-*wt phenotype in both lower grade glioma (LGG) and GBM. *C1RL*- associated genes were mainly enriched in biological processes related to the immune response. *C1RL* expression was also correlated with reduced tumour purity and increased M2 macrophage infiltration. Higher *C1RL* expression predicted unfavourable survival in patients with glioma and therapeutic resistance in GBM.

**Conclusions:**

Our results imply that *C1RL* is involved in immunological activities and is an independent unfavourable prognostic biomarker in patients with glioma. *C1RL* is a potential clinical immunotherapeutic target for glioma treatment in the future.

## Background

Glioblastoma (GBM; WHO grade IV) and lower grade glioma (LGG; WHO grade II and III) are incurable brain tumour. Existing therapeutic strategies only prolong the survival of glioma patients to a limited extent. Patients with glioma eventually die from tumour recurrence, even with aggressive treatment. Novel therapies that have been successful in other tumours, such as *PD-1* inhibition [1] and bevacizumab administration [2, 3], have failed to extend the overall survival time of patients with glioma. Tumour treating fields (TTF), a novel therapy that was recently approved for GBM treatment by the Food and Drug Administration (FDA), is not widely used in clinical practice because of its high price and difficult process [4, 5]. The current poor situation pushes us to explore the mechanism of glioma development and identify novel therapies.

The immunosuppressive microenvironment significantly contributes to the progression and therapeutic resistance of glioma. On the one hand, glioma cells induce a relatively weak immune response and enhance immunosuppression. Compared to other malignancies, glioma exhibits a lower mutational burden and fewer infiltrating T cells [6]. GBM cells block T cell activation and proliferation in response to T cell receptor stimulation by generating extracellular vesicles carrying *PD-L1* [7]. Glioma cells promote the expression of *PD-L1* on macrophages derived from healthy donors [8, 9]. Intratumoural immunosuppressive education by glioma also contributes to the rise of systemic immunosuppressive myeloid-derived suppressor cells (MDSCs) [10]. On the other hand, the brain provides an immunosuppressive environment for glioma. Compared tomelanoma in the flank, melanoma in the brain contains fewer CD8 T cells [11]. Moreover, antigen-specific cytotoxicity is systemically impaired in mice with brain melanoma [11]. Naïve T cells are sequestered in large numbers in the bone marrow in cancer patients. This phenomenon characterizes a variety of tumours only when the tumours are located in the intracranial compartment [12].

Complement C1r subcomponent like (*C1RL*) was found to be a prognostic marker in hepatocellular carcinoma [13] and renal cell cancer [14]. A gene-based analysis showed significant associations between non-Hodgkin lymphoma or diffuse large B-cell lymphoma and the *C1RL* gene [15]. *C1RL* also mediates the progression of Burkitt’s lymphoma [16]. *C1RL* is a protein-coding gene associated with ovarian adenocarcinoma and leucorrhea. In terms of molecular function, the *C1RL* protein, which is homologous to C1r, is identified as the active form of serine hydrolase [17]. The *C1RL* protein cleaves prohaptoglobin in the endoplasmic reticulum [18]. In addition, pro-C1s is proteolytically cleaved into two fragments with sizes identical to those of the two chains of active C1s by the *C1RL* protein [19]. However, the immunological and clinicopathological characteristics of *C1RL* in glioma remain unclear.

In the present study, we employed 2120 glioma specimens and 23 non-tumour brain tissues from 5 datasets to explore the clinicopathological and biological characteristics of *C1RL* in glioma. The clinicopathological features evaluated included WHO grade, histology, GBM status, *IDH* mutation status, GBM subtype, overall survival and therapeutic resistance. The biological process enrichment of *C1RL*-associated genes was analysed to explore the biological characteristics of *C1RL*. Moreover, the relationships between *C1RL* expression and tumour purity or leukocyte infiltration were analysed.

### Methods data collection

Five datasets including transcriptomic files and corresponding clinicopathological information for patients who were diagnosed with glioma (WHO II-IV) were downloaded. A microarray dataset containing 539 samples (TCGAmic) and an RNA sequencing dataset containing 702 samples (TCGAseq) were downloaded from The Cancer Genome Atlas (TCGA; https://xenabrowser.net). A microarray dataset containing 301 samples (CGGAmic) and an RNA-sequencing dataset containing 325 samples (CGGAseq) were downloaded from The Chinese Glioma Genome Atlas (CGGA; http://www.cgga.org.cn/). A microarray dataset containing 276 samples (GSE16011mic) was downloaded from Gene Expression Omnibus (GEO; https://www.ncbi.nlm.nih.gov/geo/).

### Statistics of *C1RL* expression patterns

An unpaired t test was used in comparisons of *C1RL* expression between two groups. Ordinary one-way ANOVA (multiple comparisons) was applied to compare *C1RL* expression among three or more groups. *P* < 0.05 was considered significant.

### *C1RL*-associated gene siftings and gene ontology (GO) analyses

Pearson correlation coefficients between *C1RL* and all other genes were calculated in RStudio 1.1.453 with the cor.test algorithm. *C1RL*-associated genes were defined as genes with an r value > 0.4 in the GBM dataset (TCGAmic) and r > 0.5 in the glioma datasets (TCGAseq, CGGAmic, and CGGAseq). All the *C1RL*-associated genes were introduced into DAVID (https://david.ncifcrf.gov/) for further GO analyses. The top 10 biological process terms of the GO analysis results are listed in Fig. 2.

Moreover, the detailed correlations between *C1RL* and immunosuppressive genes (*CD86*, *LGALS9*, and *TGFB1*) are shown in Fig. 3.

### Tumour purity and leukocyte infiltration

The ESTIMATE algorithm package was used to analyse tumour purity. The CIBERSORT tool (https://cibersort.stanford.edu/) was used to evaluate leukocyte infiltration. Heatmaps were produced in MORPHEUS (https://software.broadinstitute.org/morpheus/) online. The colour shows the Z score (subtract mean, divided by standard deviation) of all the expression data. The samples were ordered according to the expression of *C1RL*.

### Survival analyses

The log-rank test and Kaplan-Meier survival curves were used to describe survival differences between two groups. The survival analysis of the GSE16011 dataset was conducted in R2 (https://hgserver1.amc.nl/cgi-bin/r2/main.cgi).

## Results

### *C1RL* expression was upregulated in GBM, especially mesenchymal GBM, primary GBM and *IDH1*-wt GBM

In this study, we employed 2120 glioma specimens and 23 non-tumour brain tissues from 5 datasets. The characteristics and clinical information of the 5 datasets were summarized in Table S1.

**Fig. 1:**
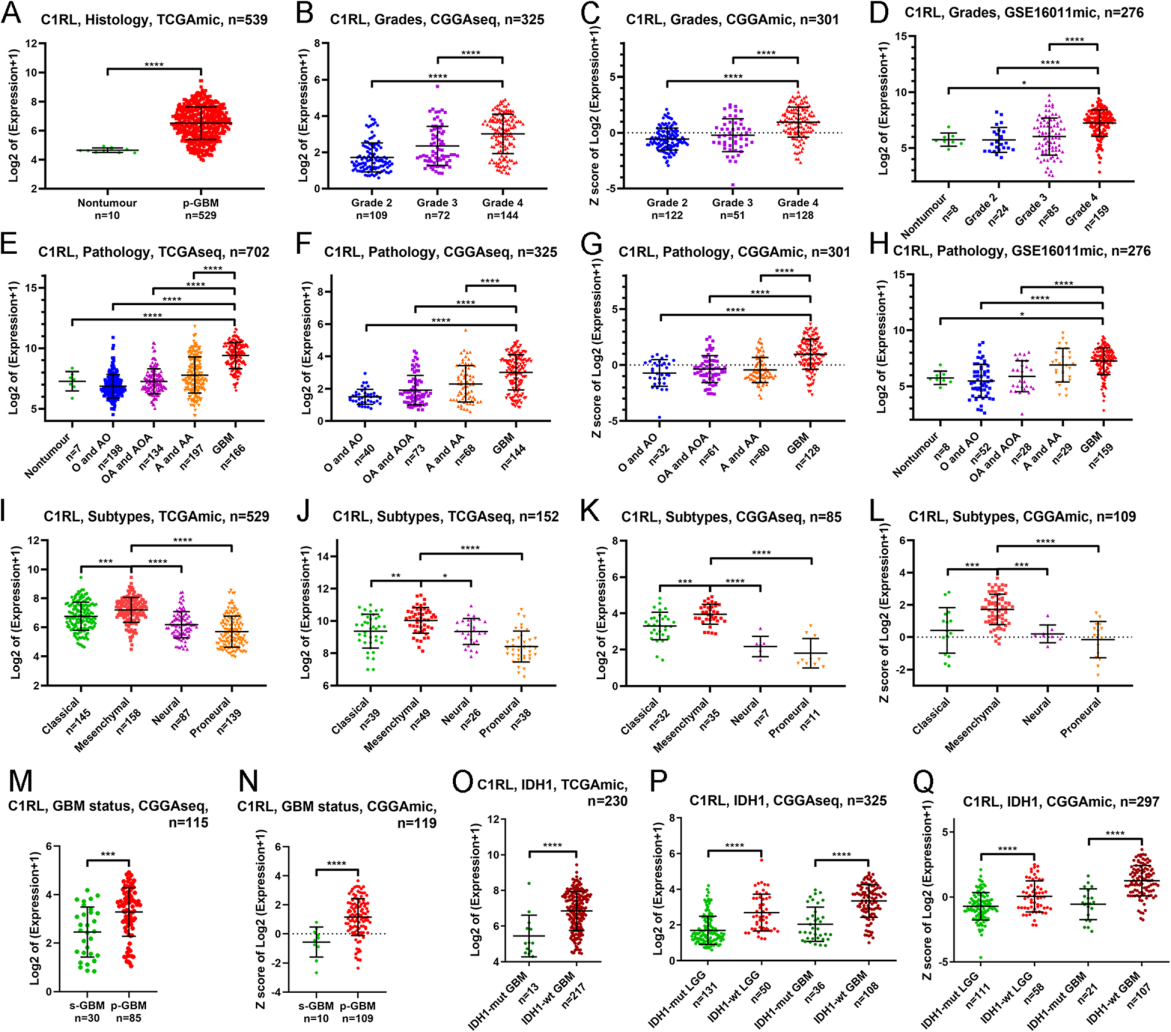
Histopathological characteristics of *C1RL* in glioma. **a**. *C1RL* expression in primary GBM (p-GBM) and nontumour brain tissues in TCGAmic. *C1RL* expression in distinct WHO grades of glioma in CGGAseq (**b**), CGGAmic (**c**), and GSE16011mic (**d**). *C1RL* expression in distinct histological types of glioma in TCGAseq (**e**), CGGAseq (**f**), CGGAmic (**g**), and GSE16011mic (**h**). *C1RL* expression in four GBM subtypes in TCGAmic (**i**), TCGAseq (**j**), CGGAseq (**k**), and CGGAmic (**l**). *C1RL* expression in samples with different GBM statuses in CGGAseq (**m**) and CGGAmic (**n**). *C1RL* expression in *IDH1-*mutant or *IDH1-*wild-type LGG or GBM in TCGAmic (**o**), CGGAseq (**p**), and CGGAmic (**q**). O, oligodendroglioma; AO, anaplastic oligodendroglioma; OA, oligoastrocytoma; AOA, anaplastic oligoastrocytoma; A, astrocytoma; AA, anaplastic astrocytoma. Mut, mutant; wt, wild-type. *, *p* < 0.05; **, *p* < 0.01; ***, *p* < 0.001; ****, *p* < 0.0001

*C1RL* expression was analysed according to the WHO classification, GBM subtype, GBM status and *IDH1* mutation status. First, the expression of *C1RL* was always highest in GBM in the 5 datasets according to both the grading system and the histology system (Fig. 1.A-H). However, the expression levels of *C1RL* in GBM samples varied greatly. Furthermore, *C1RL* expression among different subgroups of GBM was analysed. Among the four transcriptomic subgroups of GBM, *C1RL* expression was always highest in mesenchymal GBM (Fig. 1.I-L). Secondary GBM was developed from lower grade glioma and exhibited lower *C1RL* expression than primary GBM (Fig. 1.M-N). The *IDH* mutation status is a well-accepted marker for glioma classification. *C1RL* expression was higher in *IDH1*-wt GBM than in *IDH1*-mt GBM (Fig. 1.O-Q). In addition, *C1RL* expression was higher in *IDH1-*wt LGG than in *IDH1-*mt LGG (Fig. 1 P and Q). These results suggested that higher *C1RL* expression accompanies more advaced malignancy in glioma, especially in GBM.

### *C1RL*-associated genes were enriched in the biological processes of the immune response

**Fig. 2:**
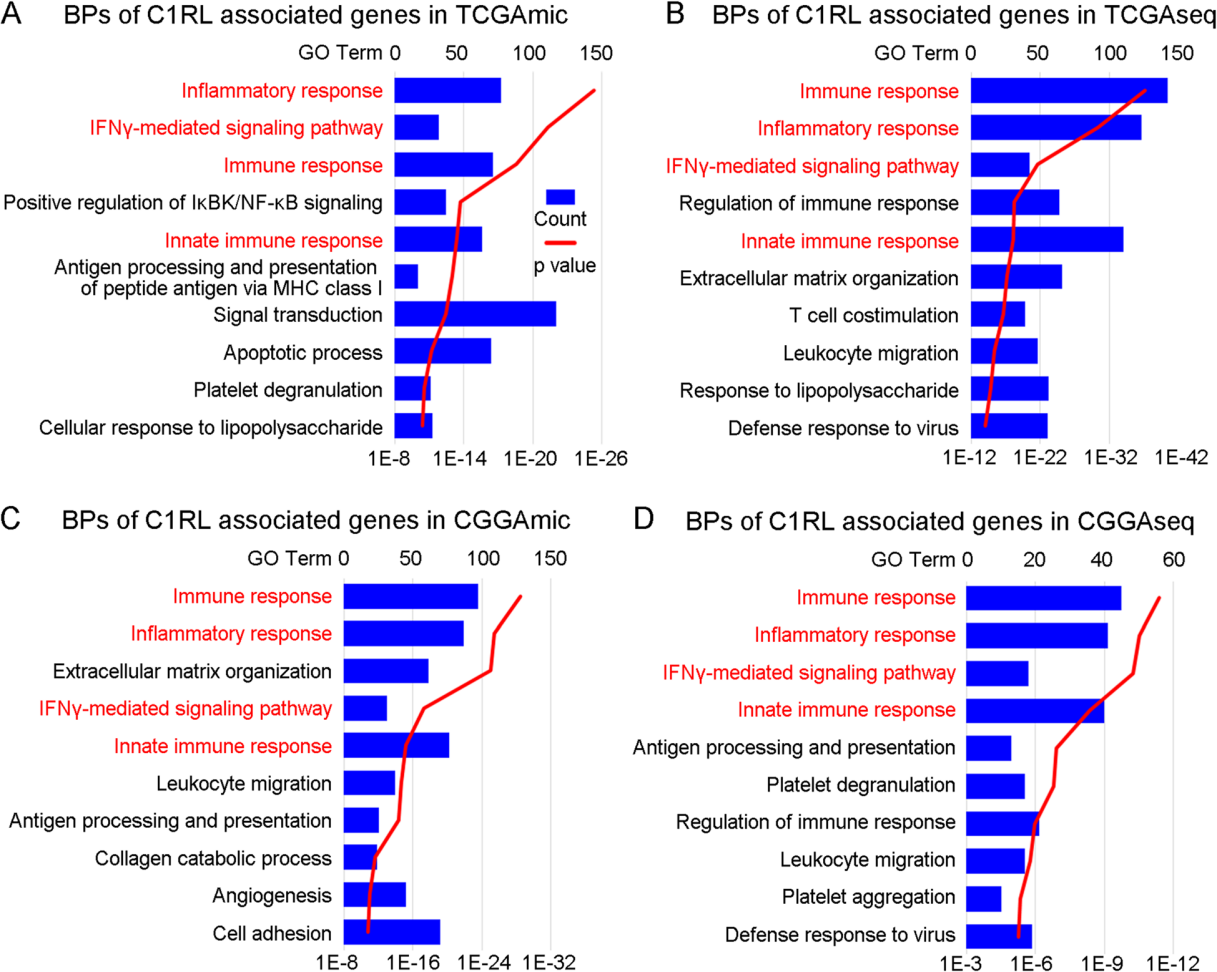
Biological processes of *C1RL*-associated genes. The top 10 biological processes of *C1RL*-associated genes in TCGAmic (**a**), TCGAseq (**b**), CGGAmic (**c**), and CGGAseq (**d**). The processes are listed in reverse order of the *p* values. The biological processes that appeared in all four datasets are marked in red

The biological function of *C1RL*, especially in tumours, has not been clarified thoroughly. Therefore, we aimed to identify the possible biological function of *C1RL* through analysis of the biological functions of *C1RL*-associated genes. *C1RL*-associated genes were defined as genes with expression trends similar to those of *C1RL* in glioma samples. All the *C1RL* genes from each dataset were listed in Table S2 and were evaluated by GO analysis. The biological processes are listed in reverse order of their *p* values. The GO analyses showed that *C1RL*-associated genes were mainly enriched in the biological processes of the immune response, inflammatory response, IFN-γ mediated signalling pathway, and innate immune response (Fig. 2.A-D).

**Fig. 3:**
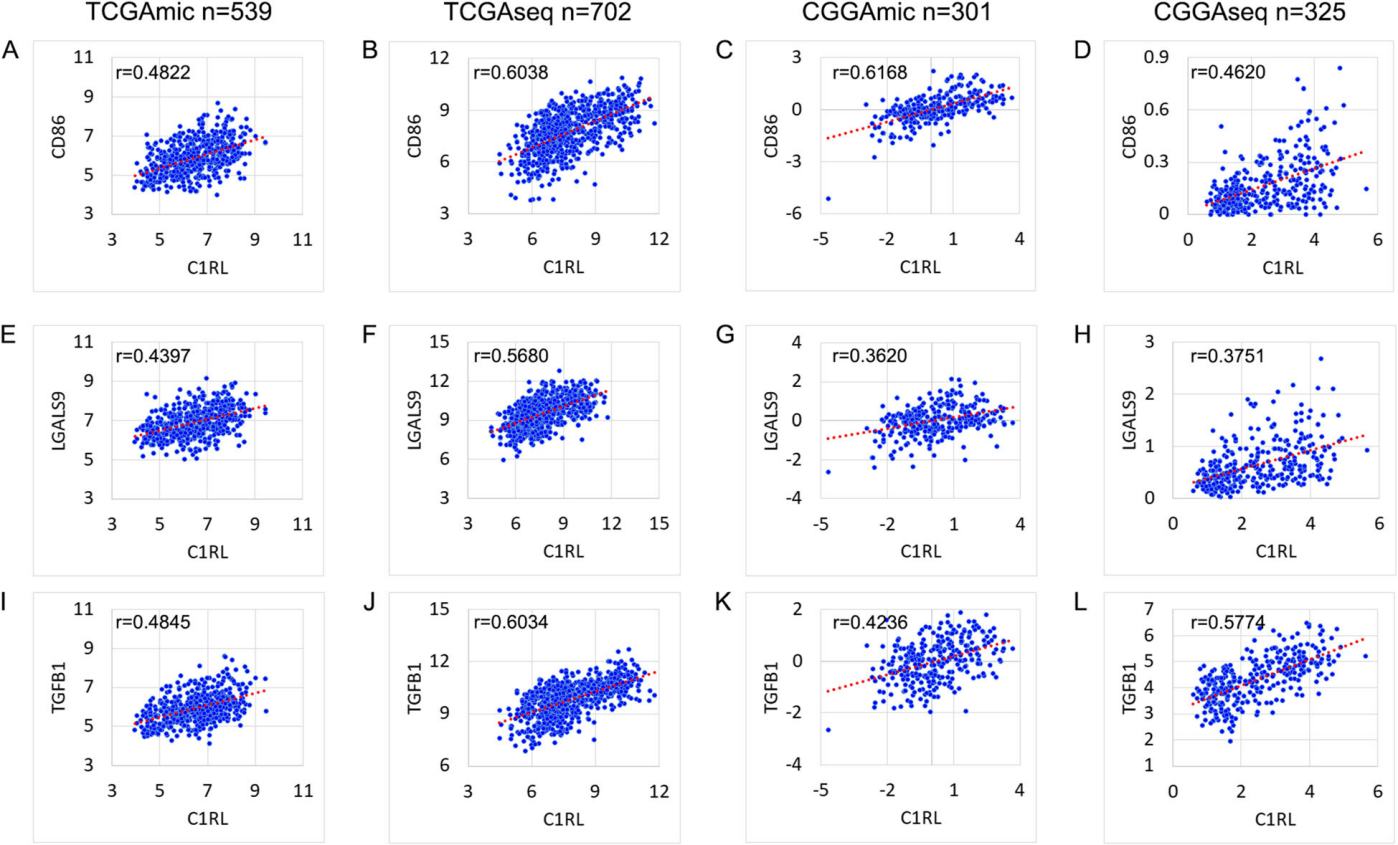
The correlations of *C1RL* with immunosuppressive genes. The correlations of *C1RL* with *CD86* (**a-d**), *LGALS9* (**e-h**), and *TGFB1* (**i-l**) in TCGAmic, TCGAseq, CGGAmic, and CGGAseq

To determine whether *C1RL* plays a positive role in the anti-glioma immune response, the expression relationships between *C1RL* and existing biomarkers were analysed. The *CD86* protein is the receptor of *CTLA4* and is mainly expressed on dendritic cells and monocytes. Galectin-9, which is encoded by *LGALS9*, was identified as the ligand of Tim-3 and plays a key role in T cell apoptosis. *TGFB1* encodes a secreted ligand in the transforming growth factor-beta (TGF-beta) superfamily of proteins. *CD86* [20], *LGALS9* [21], and *TGFB1* [22] play immunosuppressive roles in glioma. Our results showed that *C1RL* expression exhibited positive relations with *CD86*, *LGALS9*, and *TGFB1* (Fig. 3.A-L).

### *C1RL* expression was correlated with reduced tumour purity and increased M2 macrophage infiltration

**Fig. 4:**
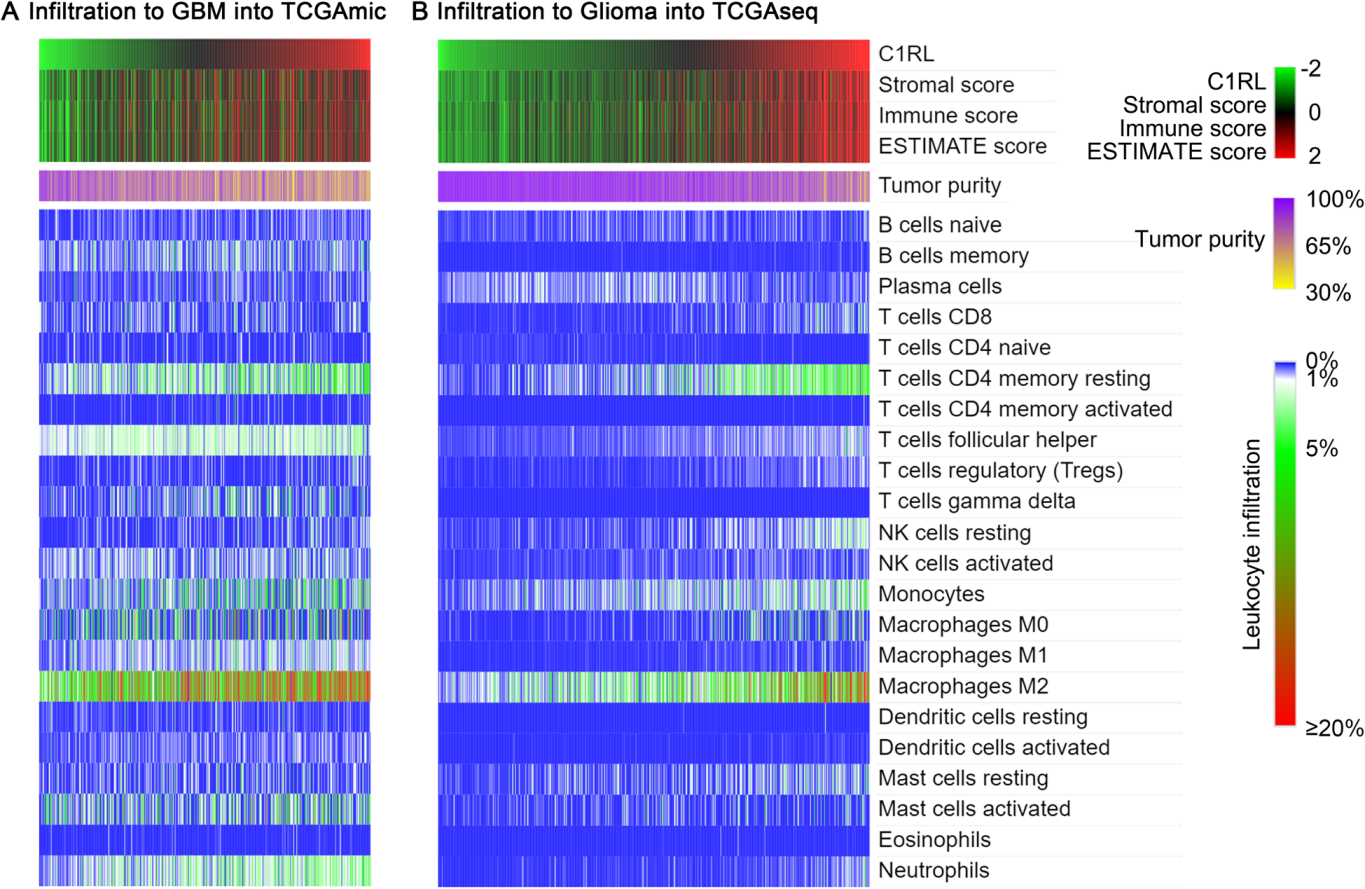
The correlations of *C1RL* with tumour purity and leukocyte infiltration. The correlation of *C1RL* with nontumour cell infiltration in TCGAmic (**a**, GBM) and TCGAseq (**b**, glioma). Samples are listed from left to right according to their *C1RL* expression level from low to high. Both the immune score and the stromal score are shown in the top panels. Tumour purity is shown in the middle panels. Leukocyte infiltration levels are listed in the bottom panels

The immune response is based on the migration of immune cells. Both tumour purity and the infiltration of 22 types of leukocytes were assessed for each sample in the TCGA datasets. The samples are displayed in order of their *C1RL* expression level. Both the immune score and the stromal score exhibited a positive correlation with *C1RL* expression trends (Fig. 4.A and B, top panels). In addition, tumour purity showed an inverse correlation with *C1RL* expression trends (Fig. 4.A and B, middle panels). Moreover, *C1RL* expression was mostly related to the infiltration of M2 macrophages among the 22 types of leukocytes (Fig. 4.A and B, bottom panels).

### High expression of *C1RL* predicted unfavourable survival and therapeutic resistance in glioma

**Fig. 5:**
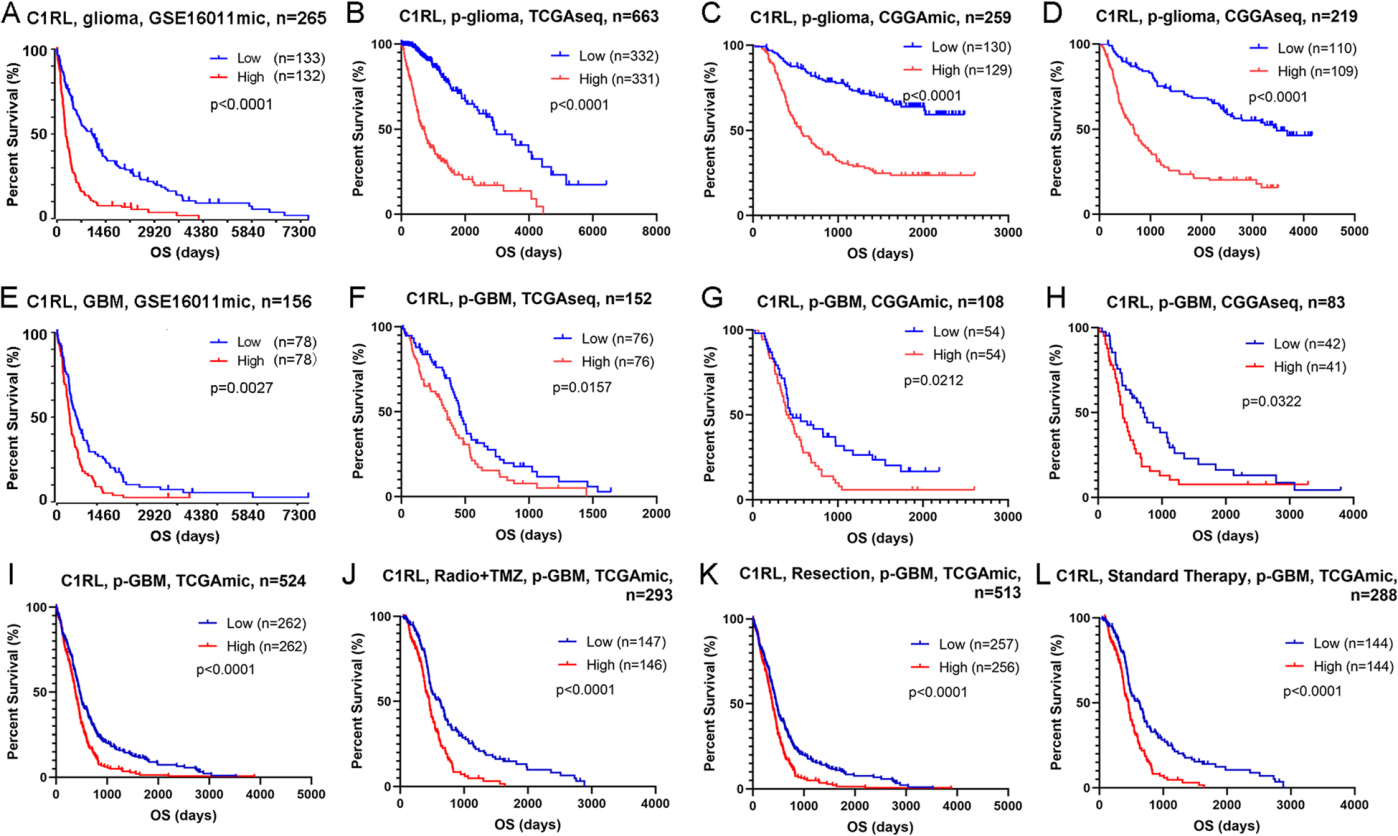
Survival differences according to *C1RL* expression in glioma. High *C1RL* expression predicted shortened overall survival for glioma patients (**a**) and primary glioma patients (**b-d**). High *C1RL* expression predicted shortened overall survival for GBM patients (**e**) and primary GBM patients (**f-i**). Primary GBM with high *C1RL* expression showed increased resistance to radiochemotherapy (**j**), resection (**k**), and standard therapy (**l**)

The median expression value of *C1RL* was used to separate samples into two subgroups. We evaluated the prognostic value of *C1RL* in the four glioma datasets. The patients with glioma exhibiting higher *C1RL* expression had significantly shorter survival times than their counterparts in the GSE16011mic, TCGAseq, CGGAmic, and CGGAseq datasets (Fig. 5.A-D). However, the histopathology characteristics in the two subgroups are significantly different (Table S3). The histopathology characteristics may contribute to the survival differences. Next, we compared survival times among GBM patients with different *C1RL* expression levels in the five datasets. All the survival curves exhibited significant differences (Fig. 5.E-I). Moreover, the effectiveness of well-accepted treatment was evaluated in different *C1RL* expression groups. The primary GBM patients with lower *C1RL* expression showed better responses to resection, radiochemotherapy (temozolomide), and standard therapy (Fig. 5.J-L). These results indicate that *C1RL* may contribute to therapeutic resistance.

## Discussion

We analysed the characteristics of *C1RL* in gliomas from various angles. According to the 2016 WHO classification of glioma, the current diagnosis of glioma is mainly based on the WHO grades and pathology and *IDH* mutation [23]. Besides, the GBM can be classified into Proneural, Neural, Classical, and Mesenchymal subtypes [24], or primary and secondary GBM [25]. The results showed that the subgroups with worse prognosis always have higher levels of *C1RL* (Fig. 1). Based on the perspectives that *C1RL* should have similar biological functions with *C1RL*-associated genes, we tried to explore the biological function of *C1RL* with *C1RL*-associated genes. The GO analyses showed that *C1RL*-associated genes enriched in immune related biological functions. But we still unsure whether *C1RL* promotes anti-tumour immune response or suppress it. Given that *CD86* [20], *LGALS9* [21], and *TGFB1* [22] play immunosuppressive roles in glioma, we further investigated the expressing relationship between *C1RL* and these immunosuppressive genes. Besides, low tumour purity [26] and high M2 macrophages infiltration [27] were reported to promotes glioma progression. So, we also analysed the relationships between *C1RL* and glioma purity and leukocyte infiltration.

*C1RL* is a negative biomarker for glioma prognosis. *C1RL* had not been mentioned in cancer until a report indicating significant associations of non-Hodgkin lymphoma and diffuse large B-cell lymphoma with the *C1RL* gene in 2012 [15]. In recent years, *C1RL* has been reported to be a prognostic biomarker in hepatocellular carcinoma [13] and renal cell cancer [14]. Our results showed evidence that *C1RL* is highly expressed in glioma samples and predicts a poor prognosis. GBM is WHO grade IV glioma and has the worst prognosis of glioma types, with a median overall survival time of 14.6 months [28]. *C1RL* expression was always higher in GBM than in LGG (Fig. 1.A-H). Unsupervised transcriptomic analysis revealed that of the GBM subtypes, mesenchymal GBM has the worst survival [29]. *C1RL* expression was higher in mesenchymal GBM than in other GBM subgroups (Fig. 1.I-L). Secondary GBM progresses from LGG within 5–10 years of diagnosis and is accompanied by a better prognosis than primary GBM [25]. Secondary GBM exhibited less *C1RL* expression than primary GBM (Fig. 1.M and N). Patients with *IDH1-*mut glioma have a better outcome than those with *IDH1*-wt glioma [30]. Relatively low *C1RL* expression was found in both *IDH1*-mut LGG and *IDH1-*mut GBM (Fig. 1.O-Q). *C1RL* not only predicts more advanced malignancy but also worse overall survival in glioma. Due to the distinct outcomes of the glioma subgroups, the differences in *C1RL* expression in different histopathological subgroups (Fig. 1.A-H) may contribute to the observed differences in survival. We further investigated survival differences between the high *C1RL* expression group and the low *C1RL* expression group in GBM and even primary GBM. The results confirmed that the expression level of *C1RL* was a survival indicator in primary GBM (Fig. 5.F-I). Resection following chemoradiation is a well-accepted strategy for primary GBM patients. Considering the effects of variant therapies, *C1RL* may play a role in therapeutic resistance (Fig. 5.J-L). Overall, our results indicate that *C1RL* is a biomarker of poor outcomes in glioma patients.

*C1RL* probably plays an important role in glioma immunosuppression. The *C1RL* protein is confirmed to be an active form of serine hydrolase [17] and cleaves prohaptoglobin and pro-C1s into their active forms [18, 19]. On the one hand, due to the suppression of lymphocyte function by haptoglobin [31], *C1RL* may modulate immunosuppression in glioma by releasing active haptoglobin. On the other hand, the association of C1s with C1r and C1q, following ligand recognition, triggers the activation of the classical complement pathway [32]. C1q plays a fundamental role in the pathogenesis of glioma [33]. *C1RL* may trigger the classical complement pathway by activating C1s and thus contribute to the pathogenesis of glioma. In addition, accumulated evidence shows that *C1RL* expression is upregulated during inflammation [34, 35]. GO analyses of *C1RL*-associated genes revealed that they were mainly enriched in the biological processes of the immune response, inflammatory response, IFN-γ mediated signalling pathway, and innate immune response (Fig. 2.A-D). Furthermore, *C1RL* exhibited positive correlations with immunosuppressive markers (Fig. 3.A-L).

*C1RL* expression was correlated with leukocyte infiltration, especially M2 macrophage infiltration. Tumour purity was proposed as an important factor in glioma. Low purity cases were independently associated with poor prognosis [26]. Glioma evolution is associated with immunological changes in the microenvironment [29]. M2 macrophages promotes glioma growth [27]. The ESTIMATE algorithm is a well-accepted method to predict the tumour purity in genomic and transcriptomic studies [36, 37]. Besides, CIBERSORT algorithm, also known as in silico flow cytometry, was developed to accurately assess the infiltration of many leukocyte subsets in bulk tumour samples, along with a signature genes file that enumerates the genes that define the signature expression profile for each immune cell [38]. The CIBERSORT algorithm can be access online (https://cibersort.stanford.edu/) to characterize cell composition of complex tissues from their gene expression profiles. In this study, both ESTIMATE algorithm and CIBERSORT algorithm were used to further assess the relationships between *C1RL* mRNA expression and 22 different immune cell populations. Increased amounts of immune cells, especially M2 macrophages, migrated into glioma tumours with relatively high *C1RL* expression (Fig. 4.A and B). All these results are consistent with the hypothesis that *C1RL* palys an immunosuppressive role in glioma.

## Conclusions

In conclusion, we analysed the immunological and clinicopathological characteristics of *C1RL* in 2120 glioma patients from five datasets. The results indicate that *C1RL* is a negative biomarker for the patients with glioma. Furthermore, *C1RL* probably plays an immunosuppressive role in the pathogenesis of glioma by triggering the activation of haptoglobin and C1s.

## Supplementary information

**Additional file 1 Table S1**. Clinical information of 2143 patients from the different datasets.

**Additional file 2 Table S2**. C1RL associated genes.

**Additional file 3 Table S3**. C1RL and WHO grade and glioma histopathology.

## Data Availability

The microarray dataset of 539 samples (TCGAmic) and the RNA sequencing dataset of 702 samples (TCGAseq) were downloaded from The Cancer Genome Atlas (TCGA, https://xenabrowser.net). The microarray dataset of 301 samples (CGGAmic) and the RNA sequencing dataset of 325 samples (CGGAseq) were downloaded from The Chinese Glioma Genome Atlas (CGGA, http://www.cgga.org.cn/). The microarray dataset of 276 samples (GSE16011mic) was downloaded from Gene Expression Omnibus (GEO, https://www.ncbi.nlm.nih.gov/geo/). The survival analysis of GSE16011 dataset was conducted in R2 (https://hgserver1.amc.nl/cgi-bin/r2/main.cgi).

## Abbreviations

C1RL: Complement C1r subcomponent like
CGGA: The Chinese Glioma Genome Atlas
FDA: The Food and Drug Administration
GBM: Glioblastoma
GO: Gene Ontology
LGG: Lower grade glioma
MDSCs: Myeloid-derived suppressor cells
TCGA: The Cancer Genome Atlas
TGF-beta: The transforming growth factor-beta
TTF: Tumour treating fields
