# Supplementary material for: Immunological and clinicopathological characteristics of C1RL in 2120 glioma patients
Source: BMC Cancer. 2020 Sep 29;20:931. doi: 10.1186/s12885-020-07436-6 (PMC7526369; doi:10.1186/s12885-020-07436-6)
Supplement: Supplementary file 1 — Additional file 1 Table S1. Clinical information of 2143 patients from the different datasets. [file 12885_2020_7436_MOESM1_ESM.docx]

S Table 1. Clinical information of 2143 patients from the different datasets

|  | TCGAmic | TCGAseq | CGGAmic | CGGAseq | GSE16011 |
| --- | --- | --- | --- | --- | --- |
| Total number | 539 | 702 | 301 | 325 | 276 |
| Age  Mean (range) | 58 (10-89) | 51 (10-89) | 43 (13-70) | 43 (8-79) | 50 (11-81) |
| Gender |  |  |  |  |  |
| Male | 320 | 398 | 180 | 202 | 184 |
| Female | 205 | 297 | 121 | 122 | 92 |
| Gender unknown | 14 | 7 | 0 | 1 | 0 |
| KPS  Mean (range) | 77 (20-100) | 84 (40-100) | Unknown | Unknown | Unknown |
| Non-tumor | 10 | 5 | 0 | 0 | 8 |
| LGG | 0 | 530 | 173 | 181 | 109 |
| O and AO | 0 | 198 | 32 | 38 | 52 |
| OA and AOA | 0 | 134 | 61 | 77 | 28 |
| A and AA | 0 | 197 | 80 | 66 | 29 |
| IDH1 wt LGG | 0 | Unknown | 58 | 48 | Unknown |
| IDH1 mut LGG | 0 | Unknown | 111 | 133 | Unknown |
| IDH1 unknown LGG | 0 | Unknown | 4 | 0 | Unknown |
| GBM | 529 | 167 | 128 | 139 | 159 |
| Primary GBM (p-GBM) | 529 | 153 | 109 | 85 | Unknown |
| Recurrent GBM (r-GBM) | 0 | 13 | 9 | 24 | Unknown |
| Secondary GBM (s-GBM) | 0 | Unknown | 10 | 30 | Unknown |
| Classical p-GBM | 145 | 42 | 15 | 32 | Unknown |
| Mesenchymal p-GBM | 158 | 55 | 70 | 35 | Unknown |
| Neural p-GBM | 87 | 28 | 8 | 7 | Unknown |
| Proneural p-GBM | 139 | 39 | 16 | 11 | Unknown |
| Subtype unknown p-GBM | 0 | 3 | 0 | 0 | Unknown |
| IDH1 wt GBM | 228 | 126 | 107 | 98 | Unknown |
| IDH1 mut GBM | 13 | 9 | 21 | 41 | Unknown |
| IDH1 unknown GBM | 288 | 32 | 0 | 0 | Unknown |
