# Supplementary material for: Immunological and clinicopathological characteristics of C1RL in 2120 glioma patients
Source: BMC Cancer. 2020 Sep 29;20:931. doi: 10.1186/s12885-020-07436-6 (PMC7526369; doi:10.1186/s12885-020-07436-6)
Supplement: Supplementary file 2 — Additional file 2 Table S2. C1RL associated genes. [file 12885_2020_7436_MOESM2_ESM.docx]

S Table 2. C1RL associated genes

| TCGAmic  n=963 | NUP98, ARTS-1, UGCG, ITGA2, SWAP70, PHLDA3, HRH1, GRN, CHST2, ITGAM, ATP1A1, CRTAP, AIM1, LTBP3, CFI, CFB, HAMP, MR1, SYPL1, IGFBP4, CFLAR, CLIC4, TMEM140, TMEM149, tcag7.1314, KIAA1199, FER1L3, M6PRBP1, RAC2, VCL, LILRB1, SRPX2, MBNL1, ADCY7, PTPN18, DKFZP586H2123, CH25H, CSF2RB, NDRG1, HLA-DQB1, MRC2, TIPARP, MYO1F, MYO1E, MYO1C, LGALS9, LGALS3, LGALS1, PLAUR, KIAA1033, LOX, ALOX5, PIGB, PIGT, TAX1BP3, PTGS2, OGFRL1, SLC39A14, ARHGAP25, ARHGAP29, NPC2, NPC1, IL4R, CAPG, PLSCR4, PLSCR3, PLSCR1, FUCA1, FCER1G, ZFP36L1, ZFP36L2, PKD2, NUAK2, CYP1B1, VPS13C, EVI2B, GNA15, OGDH, HSP90B1, CCL20, MAP3K8, DNAJC3, RALBP1, MAP3K6, COLEC12, ST5, CTSD, P2RY5, CTSZ, LIF, PKM2, FLJ13236, HLA-DRB1, CXCL2, PALMD, APOL6, TBC1D1, SMPD1, TTC26, IL6R, SLC22A18, ACOX2, MCL1, MTHFS, LMNA, C9orf167, FLVCR2, FKBP15, FKBP10, FBXO17, PLA2G5, ERBB2, SHC1, FPR1, TMEM109, SMPDL3A, AHNAK, FAM62A, SLC43A3, TMEM112B, PPP1R15A, UBE2Z, GBE1, C1S, DENND2D, NLRX1, GLIPR1, PCOLCE, RETSAT, IL10RB, DRAM, GLB1, ADAM12, PYCARD, PCSK5, PLTP, CDKN1A, TMEM127, GNS, MBOAT5, S100A9, S100A8, CYP27A1, LGALS3BP, PPP1R13L, CTBS, GNG12, IFITM3, IFITM2, SEC24D, SEC24A, TRIP6, COPZ2, STBD1, STAB1, MGC14376, AP1B1, TXLNA, HRASLS3, PSCDBP, SP100, ACADVL, AXL, SECTM1, SKAP2, CLEC7A, HLA-DPA1, IL13RA1, CTSL1, PLA2G2A, CYFIP1, LOH11CR2A, BCL3, PLK3, OAS3, PLEKHQ1, TRIM22, SERPINB1, SERPINB6, HLA-G, RIN3, RIPK1, ACTN1, ACTN4, PTPRC, LASS2, CPD, TFE3, TM9SF4, ERLIN2, HGSNAT, PLP2, CLEC5A, TM9SF1, C10orf10, NAT1, TNFAIP8, TNFAIP6, SIPA1L1, LTBP2, CLIC1, SLC35F2, MBD4, CMTM6, REXO2, LTBP1, GCC1, IRF1, LY75, CYBB, CYBA, HMHA1, CIDEB, SLA, NOTCH2, C1RL, GUSB, ARAF, TNFRSF14, B4GALT4, TNFRSF1B, TNFRSF1A, PTPN9, PTPN6, FOSL2, S100A4, CD63, GGTLA1, TPD52L2, S100A6, HSPB1, TEX261, ERBB2IP, RFTN1, CSNK1D, KCTD12, ATP13A3, H6PD, TRIP4, STOM, PDXK, TXNDC15, SREBF1, LRP10, GPC4, AGA, PIK3CG, TMEM51, TAGLN, PARP12, MAF, MGST2, BACH1, COL1A2, TGFBI, RPS6KA1, GANAB, IRF2, FYCO1, CLIP1, MNDA, AP3B1, CD44, RAB20, RAB21, CALU, CHPF, NTAN1, FILIP1L, PFN1, TRIM5, GAS6, ETF1, SIPA1, TGFBR2, FYB, NAGLU, RUFY1, FCGR2B, FCGR2A, PTGS1, DAP, PAM, CUL7, TRIM21, ARHGAP1, HMOX1, ZYX, ECM2, CALCOCO2, RGS1, CXorf9, SH2B3, PTP4A2, C1orf38, IFI35, IFI30, CD151, MFSD1, FLJ20273, CCDC109B, FZD1, FZD2, FZD7, PLCD1, LPXN, MCC, CSGlcA-T, CENTA2, PPCS, SDF4, SLC38A6, MDFIC, CXCL14, RAB27A, TBC1D2B, GFPT2, ACP6, LOC93349, SLC2A10, HERPUD1, NOD1, SLC24A6, IL17RA, MXRA8, MAN1C1, CYB561, CSTB, BST2, PSCD4, GIMAP4, LRRFIP1, RAP2B, LIMS1, ARSJ, PRKCD, CTSO, CTSA, CTSB, CTSC, CTSS, CXCL3, SYK, PRRX1, BECN1, DCTD, PDPN, BMP2K, GPR65, PLXND1, IL8, SAT1, BIRC3, FMO4, TIMP1, PARN, LYZ, LYN, TLR2, TLR3, TLR1, TLR5, ZNF217, HLA-C, SLC7A7, HLA-A, NFKBIA, HLA-F, HLA-E, MYD88, VAV1, TNFAIP3, TNFAIP2, SDCBP, RAB8A, SCPEP1, SIL1, WWTR1, SYNGR2, CTDSP1, PLOD2, PLOD3, DNASE2, UNC45A, TPK1, TANK, SLC39A8, SLC39A7, REL, CREG1, HSPB6, NRP1, ARSA, HEXA, HEXB, FBLN5, DYNLT3, ACTB, PROS1, BLVRB, SH3TC1, MYH9, TMEM2, GDPD2, TRAM2, TGFB1, TGFB2, TGFB3, F13A1, MGAT1, GLRX, VAMP5, TSPO, LRRC2, TNIP1, PLD2, DOCK2, TNFRSF12A, SLAMF8, RHBDF2, PPIC, CORO1B, FAS, FAH, C7orf42, NOD2, MAN2B2, MAN2B1, FSTL1, FLII, LITAF, PI3, KCNMB1, UBD, UBC, PLXNB2, VCAM1, TAPBPL, NAGA, CREB3L2, PVRL2, HEBP1, PLEC1, TNFSF12, DIRAS3, CROT, LSP1, APOL2, APOL1, ITGB5, ITGB4, LCP2, LCP1, LOC26010, FGR, SLC12A7, NEO1, EXT2, LY96, ORAI3, MAOB, ADORA3, ASL, COL5A2, COL5A1, LAMP2, LAMP1, ASAHL, GADD45A, GADD45B, TAGLN2, HLA-DPB1, PARP3, SH3BGRL3, SLCO2B1, MKNK1, CCR1, CD81, CD86, LRP1, GALNT10, LIMK2, MAP4, TEAD3, PLEKHF1, LAT2, GSN, ECGF1, HK2, FN1, CTNND1, MAPKAPK2, C3AR1, SAMD9, KIAA0494, TICAM1, POLD4, CECR1, FES, CD164, MSN, LEPREL1, SEC31A, TMEM123, ABCC3, GSTK1, SSPN, LAPTM5, B2M, HSPA5, SPTLC2, ITGA5, LILRB3, LILRB2, TRAM1, NADSYN1, BIN2, DPYD, HLA-DMA, CARD9, TRIM6-TRIM34, CD300A, IL10RA, TK2, MEF2A, CIB1, FVT1, MTMR11, UPP1, POR, ST3GAL1, GALNS, SLC16A3, SLC16A4, WIPF1, PTAFR, SLC15A3, C3orf64, PTPN2, DDR2, NNMT, CYBRD1, C3, C2, HMGCL, RPS6KA3, CP, CYB561D2, GAL3ST4, P4HB, PPP1CB, COL6A2, DENND3, NUCB1, SRPR, SOCS3, IL1R1, ITPR2, LAP3, CD302, NFKB1, DNPEP, EMILIN1, P2RX4, PBEF1, GMIP, OSBPL3, CHSY1, IL7R, CAPZB, THBD, DSE, HLA-B, ARFIP1, TRIP10, CPVL, PGCP, FADD, EDEM2, CCL2, RRBP1, CA12, SLC22A4, RAB7L1, WDR1, DNAJB1, SLC22A5, SGSH, ARPC1B, TBXAS1, SH3GLB1, CHI3L2, CANX, FOS, CAST, ADAM28, PALLD, RBMS1, LBA1, ANXA2, ANXA5, ANXA4, PYGL, C20orf23, SOD2, FCGBP, PIM1, FLJ20254, SLC35A2, TNC, BCAP31, STAT5A, KCTD9, SERPINA1, PXN, GATAD1, SYNPO, P4HA2, MMP19, HCK, TCN2, KRT8P12, SOAT1, NEDD9, WBSCR16, LTF, MICALL2, AEBP1, S100A11, IL32, DAB2, CSF1, ANXA1, EMR2, PRR13, TPST1, FAM129A, CCDC46, TRADD, ZDHHC24, SQSTM1, IQGAP1, HCLS1, CALD1, GEM, RARRES2, RARRES3, KIAA0247, FLNA, TMBIM1, CASP7, CASP4, CASP1, CASP8, FAM18B, PICALM, TNFRSF10B, NCSTN, NCKAP1L, PILRA, RAB7A, EFEMP1, EFEMP2, NCF2, NCF1, NCF4, SEL1L, EMP3, EMP1, IFNAR1, ANXA2P2, IRAK4, SERPINE1, FTL, RNASET2, LAMA2, PMP22, F3, TRIOBP, HS2ST1, ALDH3B1, CYR61, BACE2, IER3, ZNF394, LPP, APOL3, C1QA, ITGB2, GALNT2, CD74, SH3BP2, FLJ21963, RAB13, ZFP36, LOXL1, PFKL, MFSD5, WARS, SSR1, SSR3, ADPGK, SLC25A20, SLC25A24, CENTD2, GLB1L, SERPING1, ICAM1, TBC1D9B, SPATA20, GAA, NUMB, FLJ11286, SCAMP2, TSPAN4, C7orf49, THBS1, EHD4, EHD2, TMED9, ISG20, RAB36, FLJ20035, PLS3, PDIA5, PDIA4, PDIA3, CD163, SLC11A1, PDCD6IP, ITPKC, ITPKB, CSF1R, CLEC2B, FLJ22662, TCIRG1, BDKRB2, CD93, HOMER3, TRIM14, CHI3L1, MANBA, TMEM43, LAMB1, GCA, EPS8L2, AMPD3, DPAGT1, QTRTD1, LAMC1, PRDX6, SAMSN1, VASP, CD33, CD37, TMEM176B, LAIR1, IFNGR2, UEVLD, GALC, TAPBP, FCGR3B, PCYOX1, GLT25D1, KIAA0323, FAM26B, FXYD5, HFE, CREBL2, MAFB, MAFF, ACSL1, ADAM9, TRIM38, C21orf25, ERMAP, RCN1, ANKRD25, RHOG, CTNNA1, HP, ADM, JUNB, ZMYM6, CTNS, CSDA, EML3, MPP1, LHFPL2, BHLHB2, BHLHB3, F11R, DDB2, ELL2, APOBEC3G, APOBEC3C, EBI2, PRKD2, PLCG2, KLF6, HEATR2, SYNC1, IKBKB, FCGR1A, DHRS3, SLC2A5, SLC2A3, MTDH, ENG, PLOD1, DNAJC13, DNAJC10, FEM1C, DDEFL1, MS4A6A, SCIN, ACTG1, TMED10, LTBR, FMOD, PRSS23, FAM38A, SRGN, WAS, TLN1, LEPROT, RNASE2, RNASE4, RNASE6, CTR9, CCR5, LAMB2, FGF2, OLFML2B, SDC2, SDC4, ELOVL1, DUSP1, DUSP3, RRAS, C21orf62, C8orf4, AHNAK2, SQRDL, C17orf60, MS4A4A, NPL, BNIP2, RNH1, SPI1, LAPTM4A, HLA-DRA, CXCR4, MGAT4B, C9orf95, C5AR1, ADAMTS1, SP110, RAMP3, CLCF1, SSH3, PLEKHA4, TREM1, UBE1L, PHF11, FTHP1, HEG1, FMNL1, ZC3HAV1, AQP9, RNF216, APLP2, PARP4, ANG, SLC10A3, FHL3, GSDMDC1, FTH1, CD14, ZCCHC6, RENBP, C17orf62, AKAP13, FRAG1, S100A10, ACP2, CAPN1, SPAG1, OSTF1, KDELR2, KDELR1, PTX3, BTN3A3, STEAP3, HDAC7A, GCLM, OSMR, DPY19L1, MYO7A, FKBP5, WIPI1, GM2A, TEGT, FLNC, RCAN1, MVP, YAP1, MGC15523, ABCA1, MCFD2, KIF13B, HPS5, NFE2L2, NFE2L1, ZNF267, FCGRT, YIPF1, CYB5R1, TETRAN, DERL2, CD97, NMI, CHPT1, CSTA, GMPPA, RAD50, CEBPB, CEBPD, MAP3K14, TPP1, TGOLN2, TGIF1, BGN, MYL9, TWSG1, FNDC3B, GBP2, GBP1, MAN2A1, CD4, APH1B, STAT3, FLJ20699, MAP2K3, SUCLG2, SLC2A4RG, ABCC1, BTN3A2, CD2AP, COL8A2, GLUL, IFT122, GYS1, STT3A, NFIC, RGS19, SLC33A1, ELF1, ELF4, LDHA, PLAU, PTRF, SERPINH1 |
| --- | --- |
| TCGAseq  n=2122 | PMM2, SPN, FAM183A, SPPL2A, TGFBR2, STK40, ATP2A3, ITGA1, ITGA2, ITGA3, ITGA4, ITGA5, ITGA7, SWAP70, RARRES1, PHLDA2, GAP43, FBXL13, COL4A2, COL4A1, SLC35D2, CHST2, CHST6, BCL2A1, ITGAL, ITGAM, SERPING1, TCEA3, SIPA1, CRTAM, ATP1A1, SPHK1, IL10RA, PDIA5, ATG4A, LRGUK, CFH, CFI, CFB, ATP6V0E1, HAMP, MR1, CNPY4, AAGAB, TMEM179B, GGCX, STAC3, TTC7A, OSTM1, RUNX2, IGFBP2, IGFBP3, IGFBP7, IGFBP4, IGFBP5, CKLF, STK17A, IFI16, CEPT1, RTCD1, CLIC4, TMEM147, ASGR2, RIT1, PKIB, TMEM149, NUMBL, HLA-DPA1, FER1L4, VWA1, ZAP70, RAC2, LILRB3, NSUN7, TBC1D1, PAMR1, ERGIC1, CPPED1, GSDMD, PLK3, HRH1, PTPN12, MYCBP, HOXA6, HOXA5, HOXA4, HLA-DQB1, HOXA3, HOXA2, HOXA1, MRC2, LPAR6, MYO1G, MYO1F, MYO1E, MYO1C, GRN, HOXA10, LGALS9, LGALS8, LGALS3, LGALS1, SMS, PLAUR, LOX, LOC440957, PIGB, PIGC, PIGK, MGST2, PDK3, SEC61A1, PLVAP, ARHGAP27, ARHGAP25, ARHGAP29, SYDE1, C1orf38, FEM1C, SPRY2, CAP1, NPC2, TNFSF12-TNFSF13, IL4R, C3orf52, EFHC2, CAPG, ETHE1, PLSCR1, HLX, FCER1G, BTG3, SLA2, ZFP36L2, C15orf48, FHOD1, GJC1, PRKACA, NUAK2, C14orf119, TNFSF10, HDHD3, TMEM165, SERPINA5, EVI2B, WDR1, GNA15, UPP1, LOC284276, BCAT1, PIPOX, HSP90B1, CCL20, ALPK1, ALPK3, C8orf48, FAM70B, FSIP1, DNAJC3, MAP3K6, RAP1B, RAP1A, FAM91A1, CD200R1, CTF1, TRPV4, RARRES2, IL2RA, IL2RB, IL2RG, RARRES3, C16orf54, BAX, PRKCDBP, SLC37A2, GPR120, P2RY8, FBXO17, LIF, NCF1, LRRC42, B3GNT5, B3GNT7, B3GNT8, HLA-DRB1, XAF1, HLA-DRB5, CCDC152, FN1, SYK, MARCO, TRIM5, HFE, PALMD, ANGPT2, ANGPT1, C1orf54, MPZL2, ICMT, KRT75, ADAM33, SASH3, LRRFIP1, TTC26, SLC22A18, CFD, ITGB3BP, GAL3ST4, ARHGDIB, NANP, LMNA, HLA-DMB, DCTD, FKBP10, FKBP11, PDPN, C9orf167, FPR1, FPR2, FPR3, TMEM109, TMEM107, VSTM1, TRANK1, FKBP1A, STAT6, FBLIM1, RPN1, FRMD8, ZSCAN5A, SIX5, CLEC18A, SLC15A4, NMNAT1, ACSS3, SLC43A3, PPP1R15A, TREML3, KLF17, GBE1, ZBTB7B, HDAC1, THBS1, RPA3, C1S, C1R, COQ2, RREB1, DENND2D, SYPL1, STAT3, PRF1, LOC400804, H2AFJ, SRGN, TMEM159, C7orf31, LHFP, PCOLCE, CD300C, ACTR3, EOMES, PTGFRN, ICOS, HHEX, SAA2, SAA1, ZNF816A, ADAM12, TNFRSF11B, HUS1, UCP2, ZDHHC12, PCSK5, DUSP23, OSR2, ZNF552, COL15A1, PLTP, C20orf30, NFIL3, EN1, CABP4, FBXO39, SAT1, MBOAT1, S100A3, RPS2P32, C1orf113, LGALS3BP, PLEKHO2, C2orf29, C2orf28, EPHX3, OSTF1, CTBS, GNG10, GNG12, IFITM3, IFITM2, SEC24D, RNF19B, MAGT1, FHAD1, LAMB1, LAMB2, C21orf7, COPZ2, TGFB1, G0S2, TMEM8A, RHOQ, MGP, TGFB3, PLA2G15, TNFSF14, NUDT19, IFI6, STAB1, ECE1, CLIC1, EVC2, TXLNB, CDC42EP5, RAB36, ABI3, RHOH, OTP, SP100, DHDDS, CAV2, CAV1, TIMP1, IL12RB1, LDHA, ACOT9, RAB42, RAB43, PDLIM4, PTPLAD2, C12orf5, PDLIM1, CLEC7A, ESPNL, IL13RA2, IL13RA1, CD274, CD276, CTSL1, YY2, PPCS, NAMPT, IL15, PLA2G2A, SLC35D1, CYFIP1, PARP15, KYNU, PXDNL, PARP12, LOC100216001, MLKL, PARP10, BCL3, NKX2-5, TRIM21, OAS1, OAS3, OAS2, YAP1, TRIM22, OASL, SERPINB1, SERPINB6, ATP8B3, ATP8B1, STC1, DES, NIPSNAP3A, DDX60L, TLR8, GNAI3, GPR141, HLA-C, SLC7A7, RIN1, LAMA2, SLC16A4, RIPK1, LATS2, DDOST, GLIPR1, TLR3, HM13, ACTN1, ACTN4, CLP1, PTPRC, LASS2, CPD, BANK1, SIRPG, PRSS36, DMP1, F3, MX2, MX1, P2RY10, MAP1LC3C, TWISTNB, TM9SF1, APITD1, CRIP1, PLP2, CLEC5A, HS2ST1, C10orf10, C10orf11, SPATS2L, SRPX2, NAT1, TNFAIP8, SDC1, CPSF4, LOC257358, ACAA2, SIPA1L1, PLA2G4A, MBD4, MBD2, CMTM3, CMTM6, REXO2, SLC25A19, SLC15A3, ZNF321, CCDC19, KLRB1, TRIM34, GNG5, ALG2, ALG3, UBE2MP1, LTBP2, LTBP1, CLDN7, CYTL1, IRF1, IRF7, IRF5, LY75, CSF2RB, INMT, PIGT, C3orf64, ADAP2, GAB3, ITK, C13orf33, SERTAD1, SPATA17, CYBB, CYBA, CMTM7, TCTEX1D1, PPM1M, TCTEX1D4, FGFRL1, CRLF3, C7orf57, RNF130, C1RL, GUSB, HLA-DQB2, 43723, ROR2, ROR1, TNFRSF19, TRMT2B, B4GALT1, FCN3, TNFRSF1B, TNFRSF1A, CYP19A1, PTPN9, ARNTL2, PTPN7, PTPN6, FOSL1, FOSL2, S100A4, NUP54, CD63, SERPINA1, CD69, CD68, TPD52L2, S100A6, AOX1, GPR55, C1orf212, HSPB6, HSPB1, NCRNA00152, TEX261, PLOD2, CMPK2, KCNE3, FKBP9L, LDLRAD2, TICAM2, RFTN1, HTATIP2, PMS2L2, EHBP1L1, TNFSF13B, GLIS3, CYP2S1, GPBAR1, VIM, TGM5, TUBA1C, TMEM106A, IL10RB, G6PD, AIF1, PDCD1, DSG2, ERP44, TXNDC17, STAC, TMEM26, SPINK8, TXNDC12, DAP, LRP10, CUBN, SEC22B, NIPA2, AGA, C13orf18, GPR183, PIK3CD, PIK3CG, RER1, TMEM51, TAGLN, LOC541471, PARP14, KTI12, ERI1, MAK, ALDH3A1, MPV17, RPS6KA1, RPS6KA3, NPHP1, ARF6, ARF4, IRF2, PQLC3, RAB11FIP1, LRRC25, FAM176B, IFIH1, RNASET2, ARL11, FRRS1, MNDA, CD48, CD44, CA13, FPGT, GCNT1, RAB20, CALR, WDR78, FAR2, WDR77, NTAN1, NQO1, FILIP1L, FAM120AOS, ELK3, C10orf55, HEXA, CREM, AIDA, PDGFRL, BET1, TPM4, ETF1, CDCP1, TGFBR1, FAM159A, IER5L, TMEM173, GLB1, PPP1CA, SH3GLB1, LOC100132707, CHPF2, RABL5, FYB, TOR1A, XCL2, OST4, FCGR2B, FCGR2C, FCGR2A, ROD1, CYTH4, MYBPH, DPH3, CANT1, CKM, PTGS1, SLFN12, SDF4, CUL7, DNAJC25, DNAJC22, ARHGAP9, ICOSLG, ZFP36, RGS19, ZYX, SLC30A5, SLA, KMO, SLC39A1, CD79B, MEOX2, GMFG, CBLN3, MYL12B, MYL12A, RILPL2, IDO1, NLRP12, ECM2, CATSPER1, RGS1, RGS3, COL1A2, LOXL3, IL1RAP, ARAP3, CATSPERB, SECTM1, KANK2, DEDD2, SH2B3, GADD45A, GPR3, CD28, IFI30, C9orf21, ULBP3, HOXC9, HOXC8, HOXC4, HOXC6, CD151, HCST, C5orf62, MFSD5, MFSD7, POLR2L, F11R, BATF2, CCDC109B, MAPK13, FZD1, FZD2, FZD5, FZD6, FZD7, RASSF5, LPXN, BCL2L12, IL1R1, ATF5, ATF3, TTC12, TOR3A, MDFIC, STK3, CSF3R, CXCL11, CXCL10, CXCL14, CXCL16, ARPC4, TMSL3, KIAA1949, ECHDC2, ACP2, GLA, TMEM71, CISH, IL1RN, PRAM1, SLC27A3, PLSCR3, ACPP, AK2, NPNT, NCKAP1L, MACC1, FCGRT, SLC24A6, C3AR1, PELO, LAP3, YIPF2, NAAA, FCHSD1, RAB33B, RUNX1, PTGER2, ADAMTSL4, CYB561, C6orf141, PTPN2, C9orf44, C9orf47, TMEM194B, TEC, PSMB10, STXBP2, PDIA3P, S1PR3, CFLAR, ARSD, RPN2, TES, HOXA7, CDK2, RAP2B, HEXB, FAM188B, ARSI, LIMS3, ARSJ, ARSK, DMBX1, BZW1, LOC154761, TYMP, ZNF436, MUL1, MYADM, MFAP2, CTSA, CTSB, CTSC, CTSD, POSTN, CTSZ, CTSS, CTSW, CXCL6, CXCL9, EMB, TMEM150A, PDZK1IP1, AP2S1, CARD9, CARD6, ERP27, PSMB8, ABCA13, UBE2A, UBE2F, UBE2Z, ILK, C20orf134, C1orf87, GPR65, IL6, IL7, IL8, GNS, DTX2, BIRC3, TMEM37, KDELR2, TMOD3, KCNK6, PABPC4L, PI4K2B, LYZ, LYN, PSORS1C1, LY9, PTX3, ZNF217, HLA-H, HLA-B, HLA-A, HLA-F, HLA-E, NFKBIZ, PSTPIP2, CRYZ, MYD88, TFEC, HIST1H2BH, HIST1H2BK, VAV1, VAV3, ADIPOR1, TNFAIP3, TNFAIP2, NRBP1, DTX3L, SILV, SDCBP, LOC401397, RAB8A, PYHIN1, C6orf165, SCPEP1, FERMT3, HOXB7, SIL1, SYNGR2, PLOD1, DNASE2, C9orf64, SSBP2, BCL10, GFI1, TAP1, MT1L, RETN, TMEM87B, MOBKL1B, SHCBP1, LST1, CHCHD7, LCTL, VNN2, GDF15, TRAPPC2P1, SLC39A8, SLC39A4, GZMA, GZMB, GZMH, GZMK, MAP4K1, LOC493754, PIK3AP1, SP6, MIR155HG, LOC283314, AP1S2, AP1S3, LOC644538, NRP1, CA9, CA3, CA2, FBLN7, FBLN5, DYNLT3, C1orf84, C1orf85, VAMP3, C1orf88, APCDD1L, C10orf125, EID3, ACTB, HSPB7, PROS1, SPI1, TIFA, BLVRB, PION, BBS12, UBASH3B, RNPEP, TSPO, TLR2, SLAMF7, SLAMF6, SLAMF1, TGFB2, C17orf91, F13A1, FCRL6, SEC61G, STAT5A, GLRX, VAMP8, MMP9, MMP7, IER5, KCNE4, C9orf89, SHMT1, MORN3, RPL39L, TPK1, SCO1, TNFRSF12A, JUB, JUN, SCNN1B, SLAMF8, TWF2, GIMAP4, GIMAP6, GIMAP2, WARS, RHBDF2, FBXO4, A2M, TLR1, SLC44A3, SLAMF9, CORO1A, MOXD1, FAS, FAP, FAH, LOC100233209, C7orf42, C14orf142, TSPAN4, TNFRSF14, MAN2B1, FSTL3, FSTL1, FLII, KCTD14, UBD, UBC, ANKRD53, RAMP3, MEFV, CARD16, MGAT1, MGAT2, EPHA2, EPHA1, PDCD1LG2, VAMP5, SEMA3F, ARL9, ZC3H12A, ZC3H12D, TRADD, ZBTB42, KCNJ15, NAGA, MFAP4, PVRL2, HEBP1, HEBP2, GYPC, SLC17A9, C19orf59, LACTB, C11orf45, TNFSF13, DIRAS3, TAX1BP3, NAALADL1, C4orf47, PECAM1, TRPV2, RAET1K, SP110, CROT, SLC35F5, CIITA, ASPN, FAM96A, FAM86B2, MXRA5, MXRA8, LSP1, C1GALT1C1, CLEC18C, HMOX1, AQP5, TMEM50A, C10orf81, APOL2, APOL1, ITGB1, ITGB3, ITGB5, ITGB4, ITGB7, LCP2, LCP1, GNPDA1, EGF, LBP, FGR, SLC12A7, EXT2, ATP1B3, LY96, ZAK, C14orf169, CCDC125, ITPKC, LGALS12, TMED2, MAOB, FABP5, ASL, COL5A2, COL5A1, 43717, LENG9, MED18, NLRC4, SLC9A1, SERPINF1, HCK, SLC30A7, TAGLN2, GJD3, HLA-DPB1, CNN3, XKR8, NOP10, EXOC3L2, TRPM8, TRPM4, TUBB6, HERC5, SH3BGRL3, PSMB9, MKNK1, CCR1, CCR2, CCR3, CCR4, CCR5, CDSN, CCR7, BCAS4, DRAM1, CD80, CD86, BCL7B, EFNA4, CD8A, GALNT12, RUNX3, HLA-DOA, HLA-DOB, ANTXR2, SSH3, TEAD2, PTPN22, BRI3, APEX2, SPOCD1, CLDN23, DMRTA2, NFAM1, HLA-DQA1, HLA-DQA2, CASP10, NBEAL2, SERPINB8, FAM19A3, HK2, HK3, CACNA2D4, GPR160, TFPI2, NR1I2, BATF, TRIM6, MAPKAPK3, MAPKAPK2, PIK3R5, PDIA4, PMFBP1, PTCRA, LRRC15, XCL1, LRG1, TNFAIP8L2, CCDC103, POLD4, CECR1, CHRNA9, MSN, LEPREL1, OPLAH, XBP1, COL8A2, COL8A1, PDGFD, GTF2E2, UBA7, SIGLEC7, LAPTM5, B2M, HSPA7, HSPA6, HSPA5, SIGLEC5, PI3, TRAPPC3, BAK1, PFN1, LILRB2, LILRB1, PPIC, PPIB, DNALI1, MEIS3P1, C19orf10, TMEM70, CDC42, BIN2, RGS22, DPYD, PLB1, PCDH18, RALB, HLA-DMA, PCK2, SYTL3, RELB, ZMPSTE24, MRPL36, CRISPLD1, CLEC17A, HSD3B7, LOC100129034, CD300A, CD300E, SHOX2, TMED5, TWSG1, SEPN1, CYP27A1, CD300LF, CD300LB, HBXIP, EVC, MTMR11, LOC285830, CELSR1, NEK6, LRRC32, C20orf195, SERPINA3, GALNS, SLC16A3, SLC16A5, ARHGAP30, LOC647946, LYPLA1, SFRP4, HOTAIR, RBM47, PRDM1, S100A11, S100A13, PPM1J, LMO2, PAX3, GINS4, NNMT, AGBL2, SUSD2, ADPRH, PILRA, GPNMB, PSMA5, C3, C2, CP, KCNJ8, AGPAT2, COL1A1, RCSD1, AGPAT9, CMYA5, TCIRG1, TFRC, SPAG4, C19orf35, PPP1CB, C11orf21, KLHL4, C11orf24, COL6A1, COL6A3, COL6A2, SPAG1, DENND3, SLC34A2, KCNMB1, NUCB1, SOCS1, SRPR, SOCS3, SOCS2, IL1R2, ABHD15, TGFB1I1, ARPC2, ARPC5, UBQLNL, IL17RC, CD302, GPN1, NFKB1, DNPEP, RAP2C, EMILIN2, EMILIN1, DAPP1, HIVEP3, FAM115C, P2RX4, DENND1C, PQLC2, FAM114A1, GRWD1, PTGR1, HSPC157, OSBPL3, ARHGAP15, ARHGAP18, IL7R, FGL2, PVT1, SPP1, THBD, DSE, HS3ST3B1, TTC38, SLC38A6, ITPRIP, RNF135, PLA2G5, CAPZA2, CAPZA1, GEMIN7, GJB2, PSMC2, PSMC4, ARFIP1, TRIP10, CPVL, PGCP, FADD, ARMC10, EDEM2, CCL2, CCL7, CSDAP1, HS3ST3A1, PLEK2, CD40, DNAJB1, TNIP1, FAM65C, RNF149, SGSH, TMEM59, CALU, ACCS, GPR132, ATP6V1C2, OCIAD2, PPP1R3B, TBXAS1, SHC1, TRAF3IP3, PNPLA4, WEE1, ATG10, TGFBI, PL-5283, TANK, LCK, PHTF1, CAST, GPR82, GPR84, IL18, IL10, IL16, PALLD, TTF2, LEPRE1, AEBP1, LTB, ANXA1, ANXA5, ANXA4, DUSP14, DUSP10, SQSTM1, MS4A7, DSC2, PYGL, MAGOH, C20orf26, C20orf24, RAB27A, FCGBP, KCNN4, FASLG, RHOA, KRT7, CCDC65, TDO2, IL1A, CLEC18B, PIM1, TAF13, SLC35A4, SLC35A2, PIK3R6, TAF12, PGM2, APLN, PMP22, SLC25A43, SLC25A45, HOXD13, HOXD10, BEST4, WWTR1, TNC, SPA17, WTAP, SERINC2, BCAP31, KCTD9, IBSP, SBNO2, PSMD9, P4HA2, P4HA3, TMEM140, IQCG, PIRT, LOC441294, MMP14, MMP11, HLA-DRA, PRG2, SP140L, MGC12916, GSX2, KCNK13, C7orf25, C7orf29, SIRPB2, SOAT1, LTF, ALG12, ALG14, RIOK3, MICALL2, PRDX4, RBMS1, TWIST1, TMEM217, TMEM214, MREG, IL32, GDF5, SNAP23, TMSB10, SAMSN1, LITAF, EMR1, EMR2, ANXA2, PRR13, FAM129A, NCF1B, ADSSL1, DNAH9, PSMD8, CCDC46, SPRY1, FAM177B, FBXO22, TBX19, SLC35C1, AIM1, RNASE2, WIPI1, IL18BP, IQGAP1, IQGAP2, NOD1, HCLS1, RNASE6, YKT6, CALD1, MET, P2RY6, CLN5, C2orf39, SIGLEC9, RINL, GEM, LAMC1, GPR171, SP140, GPSM3, ADPGK, PRELID2, HSH2D, PDE6G, LOC400696, GPX8, TMBIM4, TMBIM1, CASP6, CASP7, CASP4, CASP5, CASP3, SMC4, CASP1, CASP8, MMRN1, ALOX5, TOR1B, TEAD3, TNFRSF10C, HYAL2, CSDA, PROCR, EFEMP1, EFEMP2, OSTC, NCF2, NCF4, VDR, DEGS1, EMP2, EMP3, EMP1, MVP, TMEM111, APOB48R, TXK, LSM10, SOD3, MXRA7, TTC39A, ANXA2P1, ANXA2P2, IRAK2, IRAK4, GATA3, TTC39B, SH2D1A, RELL1, SHISA5, SERPINE1, PDLIM7, PDLIM3, FTL, ZDHHC1, PAFAH2, ZDHHC5, ANPEP, C1orf144, CAPNS1, NCSTN, MDK, ATP5EP2, C2orf18, PLAC8, PSD4, SSR3, GPR157, C13orf26, C13orf29, RBPMS, SGMS2, HOXD11, TM4SF1, FBP1, MYOF, HMGCL, ALDH3B1, CYR61, HGF, HGD, C7orf68, BACE2, IER3, ZNF468, CYB561D2, METTL7B, IFT57, APOL6, SAMD9L, APOL4, CLECL1, C1QC, C1QB, C1QA, MGAT4B, VSIG4, ITGB2, ARL4C, PLXDC2, GALNT4, GALNT5, GALNT2, GALNT3, ST8SIA4, CD74, CD70, CD72, RFC2, PSEN2, ASCL2, GAPDH, SERPINH1, IGF2BP3, IGF2BP2, LOC606724, CD101, CD109, MRPS15, CD247, MRPS12, CD248, LOXL1, LOXL2, LOXL4, TNNI2, GPR1, DCBLD2, LILRA5, CLEC4A, SMPDL3A, APOC2, BHLHE40, PYCARD, SLC25A20, GAPT, SLC25A24, BST1, BST2, KDELC2, B9D2, PGK1, FAM86C, SMAGP, WARS2, GLB1L, ULK4, SNX10, ICAM3, ICAM1, ZNF613, NEAT1, C1orf162, C2orf77, NUMB, COMMD10, SPINT1, C7orf49, EPSTI1, C9orf6, C9orf3, PSENEN, CPA4, OSM, FAM113B, TMEM50B, EHD4, EHD2, REEP4, SNX9, HMGA2, ISG20, CD52, CD53, CD58, C8orf76, RAB34, RAB32, RAB38, DOK2, DOK3, DOK1, PLS3, PDIA6, PDIA3, RAB3D, CD163, CD164, CD226, TMEM185B, MUC1, CRB2, CLEC2B, VENTX, DNASE1L1, CD40LG, SHROOM3, BDKRB1, BDKRB2, FAM20A, FAM20C, HAT1, CTHRC1, FLJ35776, RDH5, RRAS, ZNF683, EFNB2, CCRL2, CHI3L2, CHI3L1, ASB9, TBL2, KHNYN, TC2N, POM121L9P, TMEM49, TCTN1, TMEM43, MED8, ZDHHC18, ZNF600, LOC144571, GCA, TCF7, GCK, POP4, AFP, BTK, AREG, RASGRP4, LOC653653, DPAGT1, SEPHS2, F2RL2, PRDX6, CCL5, RDH10, VASN, VASP, GMPPB, LAMP3, CD33, CD37, TXNDC3, TXNDC5, FAM46A, FAM46B, TPST1, TMEM176A, TMEM176B, ORMDL2, CD3D, CD3E, CD3G, ACADS, FOXJ1, HSPG2, LAIR1, IFNGR2, ECSCR, PLIN2, HCP5, NEU1, C10orf41, DEF6, DHX58, DDIT4L, GALM, TAPBP, FCGR3A, PRICKLE3, GLT25D1, LOC400759, CALHM2, KIAA0495, HPD, OAZ1, TMED9, FAM26F, FAM26E, HAVCR2, FXYD5, PLIN3, C6orf15, PALM2-AKAP2, RBP1, ADAM6, ARPC1B, OLFML1, OLFML3, ADAM9, GPRC5A, WISP1, VRK2, ERMAP, RCN3, RHOG, CYTIP, TMEM64, TMEM61, TMEM60, TNFRSF11A, TRIM38, TREML2, S100A9, S100A8, ADM, S100A2, SH2D4A, JUNB, TRAF5, TBC1D10C, MOV10, SLC33A1, TPM3, CAMK2D, C6orf150, ANO6, POLR1D, COL3A1, WDR41, JAK3, HOXB9, HOXB2, HOXB3, HOXB6, HOXB4, PPARG, TREML1, RHOJ, CD14, PLXND1, RHOC, DYRK3, LHFPL2, ENPEP, FLNC, DDB2, MCAM, APOBEC3H, APOBEC3D, APOBEC3G, APOBEC3F, APOBEC3C, FAM109B, MSR1, NAA20, KLF6, TMEM220, HEATR2, BATF3, KLHDC7B, KLHDC7A, NUP37, FUCA1, FCGR1A, IKBKE, UNC93B1, MICB, RARS, MIPEP, GNLY, IMPA2, IL15RA, M6PR, SNAI1, OSBPL10, SLC2A9, SLC1A5, LOC100270710, TEAD4, SHKBP1, CEACAM1, CEACAM4, TMEM154, ENG, LAT2, TRNAU1AP, MOCOS, TMEM67, VKORC1, PLOD3, ELMOD2, TNFSF8, CNIH4, DNAJC10, NUDT16P1, MS4A6A, LMAN1, LMAN2, MLX, SCIN, ACTG2, SLC11A1, TMED10, LTBR, MAP3K8, MARVELD1, FMOD, FMO4, SH2D2A, PSRC1, PRSS23, LXN, STX3, TAPBPL, GBP1, FRZB, SHQ1, PKM2, BTBD19, KRT80, CD180, WAS, C5orf32, STX11, DPP4, C14orf86, COQ10B, OTOS, RNFT1, C11orf63, RNASE3, RNASE4, C14orf50, CCDC89, CD7, NECAP2, IKBIP, NOX4, TRAM1, SLC26A2, OLFML2B, OLFML2A, GCH1, SDC4, TTYH3, RILP, DUSP5, DUSP6, LRRC8E, GBGT1, OR51E1, LRRN4CL, C21orf63, C21orf62, FAM83G, C8orf4, TMEM106C, PTGIR, PKD2, C1orf91, PTPN18, GCOM1, SQRDL, TMCO4, MFSD1, MS4A4A, AHR, COLEC12, ACTA2, TSTD1, EDARADD, KIF9, KCNQ1, HOXC10, VEGFA, PTGER4, FLOT1, FLOT2, NCF1C, CCDC8, HTRA3, PHEX, S100Z, C17orf87, ARID5A, C6orf115, LAPTM4A, ALOX5AP, NLRC5, ESYT1, CXCR3, CXCR2, CXCR6, CXCR4, SERTAD3, IFI44, C5AR1, TRIP4, ADAMTS1, ADAMTS3, COTL1, CLCF1, PLEKHA4, PLEKHA9, STBD1, TRIB1, LUM, VNN1, TREM1, TREM2, MYH9, TRIP6, ZBTB8OS, PHF11, NKG7, LOC151534, RIPK3, ZMYND12, BOLA3, GPR39, YDJC, FMNL1, NEXN, AQP9, FAM126A, ADORA3, PARP9, EPHB4, TOM1L1, ANG, AK3L1, SLC10A3, SLC10A6, FHL3, ITPRIPL2, ITPRIPL1, C5orf15, FLJ10661, NADK, ZCCHC9, C17orf60, PIH1D2, S100A10, S100A16, C19orf40, CYBRD1, CAPN5, CAPN1, SOD2, P4HB, SLC2A10, KDELR3, KDELR1, CLEC12A, ST14, MOBKL2C, TCTN2, CMAH, TXLNA, PLEK, TMEM150B, ODF3L1, AGTRAP, ELL3, RNF144B, STEAP1, STEAP3, UBASH3A, GCLM, OSMR, DPY19L1, MAN1C1, MYO7A, FKBP9, NAPSB, TYROBP, MANF, SAMD9, GPR172B, A2LD1, GPR18, PANK2, TMEM22, ZNRF2, GPX1, GPX7, FLNA, TNFAIP6, RCAN1, RCAN3, PARVB, SIT1, PARVG, CCNYL1, PLCG2, RTP4, SPON2, MCFD2, NMUR1, LILRA6, FAM111A, LILRA2, UPRT, HPS1, HPS3, NFE2L3, C17orf44, PLBD1, ROBLD3, IAH1, LOC100130776, RHOD, YIPF1, OSCAR, CYB5R1, C19orf66, FTSJ2, DERL2, DERL3, PRR24, RAG1, CD99, CD96, CD97, CD93, HDAC3, HDAC7, NMI, SKAP2, CST7, SKAP1, CSTB, CSTA, GMPPA, ESM1, AMIGO2, CEBPB, CEBPA, TRAT1, CEBPD, MTHFS, CTNNA1, FAM181A, FUCA2, TGIF1, FES, BGN, MYL6, MYL9, CHSY1, FAM110C, SYNC, RGS18, LY86, SUMF2, SUMF1, MICA, FNDC3B, SURF4, GBP3, GBP2, GBP5, CD4, CD5, CD6, CD2, STAT1, PODNL1, MAP2K3, FBXO22OS, TPRG1, SIGLEC10, MAP3K14, SLC2A4RG, ABCC3, IFI35, DNAH11, ENPP1, CD2AP, SLC47A2, TP53I13, PDGFA, AGXT2L2, GYS1, PLAU, KIAA0040, STT3A, PLAT, ETV7, ETV6, TNFRSF10D, IL4I1, PLEKHG1, RGS16, BTN2A3, BTN2A2, ELF5, ELF4, PRPS2, CDR2, ACAP1, PLEKHG6, GNGT2, PTRF, GSTK1 |
| CGGAmic  n=1561 | ABCA13, ABCC3, ABHD15, ACOT9, ACOT9, ACP5, ACSS3, ACTA2, ACTB, ACTG2, ACTN1, ACTN1, ACTN3, ADAM10, ADAM12, ADAM9, ADAMTS1, ADAMTS1, ADAMTS14, ADAMTSL4, ADAP2, ADAP2, ADM, ADPGK, AEBP1, AEBP1, AFF1, AGA, AHR, AIFM2, AIM1, AK2, AK2, AKAP12, AKAP2, ALOX5, ALOX5AP, ALOX5AP, ALPK1, ALPK1, AMICA1, AMICA1, AMIGO2, ANG, ANGPT2, ANGPT2, ANGPTL4, ANO1, ANO6, ANO6, ANPEP, ANTXR2, ANTXR2, ANTXR2, ANXA1, ANXA2, ANXA2, ANXA2, ANXA2P1, ANXA2P3, ANXA4, ANXA5, AP1S2, AP2S1, APH1B, APLN, APLP2, APOB48R, APOBEC3C, APOBEC3F, APOBEC3G, APOBEC3H, APOL1, APOL1, APOL2, APOL6, AQP9, ARAP3, AREG, ARHGAP15, ARHGAP15, ARHGAP18, ARHGAP29, ARHGAP30, ARHGAP9, ARHGDIB, ARID5A, ARL1, ARL1, ARL11, ARL4C, ARMC10, ARNTL2, ARPC1B, ARSJ, ASB9, ASL, ASPN, ATF3, ATF3, ATL3, B2M, B3GNT5, B4GALT1, BACE2, BACE2, BATF, BATF3, BCAT1, BCAT1, BCAT1, BCL10, BCL2A1, BCL2L12, BCL3, BDKRB2, BET1, BGN, BHLHE40, BIRC3, BMP2K, BST1, BST2, BTN2A2, BTN2A2, BTN2A2, C10orf10, C10orf10, C10orf11, C11orf63, C12orf5, C13orf18, C13orf26, C13orf33, C15orf48, C17orf60, C17orf91, C1orf162, C1orf162, C1orf38, C1orf38, C1orf54, C1orf85, C1QA, C1QB, C1QC, C1R, C1RL, C1S, C2, C2, C20orf26, C21orf62, C21orf62, C21orf63, C21orf63, C21orf7, C3, C5AR1, C5orf32, C5orf62, C6orf115, C6orf141, C7orf42, C8orf4, C8orf4, C9orf167, C9orf21, CA12, CA12, CA13, CA13, CA3, CACNA2D4, CALD1, CALD1, CALHM2, CALU, CAPG, CAPZA1, CAPZA1, CARD16, CARD17, CARD6, CASP1, CASP10, CASP4, CASP4, CASP5, CASP6, CASP7, CASP8, CAST, CAST, CATSPER1, CAV1, CAV1, CAV2, CCDC102B, CCDC102B, CCDC109B, CCDC19, CCDC46, CCDC46, CCDC89, CCL2, CCL20, CCL26, CCL5, CCNYL1, CCPG1, CCR1, CCR2, CCR2, CCR5, CCR7, CCRL2, CD109, CD109, CD14, CD163, CD164, CD164, CD2, CD247, CD248, CD274, CD28, CD300A, CD300C, CD300LF, CD302, CD302, CD33, CD33, CD3D, CD3G, CD44, CD47, CD48, CD48, CD52, CD53, CD58, CD59, CD63, CD63, CD68, CD69, CD72, CD74, CD86, CD93, CD93, CD96, CD97, CD97, CDCP1, CDH11, CDH6, CDH6, CDKN1A, CEBPB, CEBPD, CEBPE, CEBPG, CELSR1, CFB, CFD, CFH, CFH, CFH, CFHR3, CFI, CFI, CHI3L1, CHI3L1, CHI3L2, CHPF2, CHRDL2, CHRNA9, CHSY1, CIITA, CIITA, CLCF1, CLDN1, CLDN23, CLEC18B, CLEC2B, CLEC4A, CLEC5A, CLEC5A, CLEC7A, CLEC7A, CLIC1, CLIC4, CLIC4, CLIC4, CMAH, CMAH, CMTM6, CMTM6, CMTM7, CMYA5, CMYA5, CNN1, CNN3, COL12A1, COL13A1, COL18A1, COL1A1, COL1A2, COL3A1, COL3A1, COL3A1, COL4A1, COL4A1, COL4A2, COL5A1, COL5A1, COL5A2, COL5A2, COL5A2, COL5A3, COL6A1, COL6A2, COL6A2, COL8A1, COL8A1, COL8A1, COL8A2, COLEC12, COPB2, COPZ2, CP, CPD, CPD, CPPED1, CPVL, CRB2, CREG1, CREM, CRIP1, CRLF3, CRYZ, CRYZ, CSDA, CSDA, CSTA, CSTA, CTBS, CTBS, CTGF, CTHRC1, CTSA, CTSB, CTSB, CTSB, CTSC, CTSC, CTSL1, CTSS, CTSS, CTSZ, CXCL10, CXCL14, CXCR4, CXCR6, CYBA, CYBB, CYBB, CYBRD1, CYLD, CYP1B1, CYR61, CYR61, CYR61, CYTIP, DCBLD2, DCDC2, DCN, DCTD, DDB2, DDIT4L, DDX58, DDX60L, DENND2D, DERL1, DERL2, DES, DEXI, DIRAS3, DNAH9, DNAJB1, DNAJB11, DNAJC10, DNAJC10, DNAJC22, DNAL1, DNALI1, DNASE1L1, DOK3, DOK3, DPH3, DPH3B, DPY19L1, DPY19L1, DPY19L1, DPYD, DPYD, DRAM1, DRAM1, DRAM1, DSE, DTX3L, DTX3L, DUSP1, DUSP23, DUSP5, DUSP6, DYNLT3, DYNLT3, DYNLT3, DYRK3, DYRK3, ECE1, ECHDC2, ECM1, ECM2, EDEM2, EEA1, EFEMP1, EFEMP2, EFNB2, EFNB2, EHD2, EHD2, EHD4, ELF4, ELMOD2, EMB, EMB, EMILIN1, EMILIN2, EML4, EMP1, EMP3, EMR2, ENG, ENPP1, EPSTI1, EPSTI1, ERI1, ERO1L, ERP44, ESM1, ETF1, ETV6, ETV7, EVC, EVC2, EVI2B, F11R, F11R, F13A1, F2RL2, F3, FABP5, FABP5, FABP5, FABP5, FAM111A, FAM114A1, FAM114A1, FAM126A, FAM129A, FAM129A, FAM176B, FAM181A, FAM183A, FAM20A, FAM20A, FAM20C, FAM26E, FAM26F, FAM46A, FAM46B, FAM65C, FAM86B2, FAM86C, FAM91A1, FAP, FAS, FBLIM1, FBLN5, FBLN5, FBP1, FBXO17, FCER1G, FCGBP, FCGR2A, FCGR2B, FCGR3A, FCGR3B, FCGRT, FEM1C, FEM1C, FERMT3, FES, FGFR1OP, FGFRL1, FGL2, FGR, FHOD1, FILIP1L, FKBP11, FKBP9, FKBP9, FKBP9, FKBP9L, FLNA, FLNC, FMOD, FN1, FN1, FN1, FNDC3B, FNDC3B, FOSL2, FOSL2, FOSL2, FPGT, FPR1, FPR3, FRZB, FSIP1, FSTL1, FSTL3, FTL, FTL, FTL, FUCA1, FUCA2, FXYD5, FXYD5, FYB, FYB, FYCO1, FZD1, FZD6, G0S2, GADD45A, GADD45B, GALM, GALNT10, GALNT3, GALNT4, GALNT4, GAPT, GBP1, GBP1, GBP2, GBP3, GBP3, GBP5, GCLM, GCLM, GCNT1, GCOM1, GDF15, GEM, GGT5, GGT5, GIMAP2, GIMAP4, GK, GLA, GLB1, GLB1, GLIPR1, GLIPR1, GLRX, GLT8D3, GLT8D4, GLT8D4, GMFG, GNG12, GNG5, GPATCH2, GPNMB, GPR183, GPR65, GPR84, GPX8, GPX8, GPX8, GRB10, GRN, GSDMD, GUSB, GZMA, GZMK, H2AFJ, HAMP, HAVCR2, HAVCR2, HCCS, hCG_1990547, HCLS1, HCP5, HDAC7, HDAC7, HEBP2, HEXB, HFE, HFE, HGF, HGF, HGSNAT, HGSNAT, HK2, HK3, HK3, HLA-A, HLA-B, HLA-B, HLA-C, HLA-DMA, HLA-DMA, HLA-DMB, HLA-DMB, HLA-DOA, HLA-DOA, HLA-DOA, HLA-DPA1, HLA-DPA1, HLA-DPB1, HLA-DPB1, HLA-DQA2, HLA-DQB2, HLA-DRA, HLA-DRB1, HLA-DRB3, HLA-DRB4, HLA-DRB5, HLA-DRB5, HLA-E, HLA-E, HLA-F, HLA-G, HLA-J, HOXB2, HOXB4, HRH1, HRH1, HS2ST1, HS3ST3B1, HSP90B1, HSP90B1, HSPA4, HSPA4, HSPA5, HSPA6, HSPB1, HSPB1, HSPB1, HTATIP2, HTRA3, HUS1, IBSP, ICAM1, ICAM3, IDO1, IFI16, IFI16, IFI30, IFI44, IFIH1, IFITM1, IFITM2, IFITM3, IFITM4P, IFNAR1, IFNGR2, IGFBP2, IGFBP3, IGFBP4, IGFBP5, IGFBP5, IGFBP5, IGFBP7, IKBIP, IKBIP, IL10RA, IL13RA1, IL13RA1, IL13RA1, IL13RA2, IL15, IL18, IL1R2, IL1RAP, IL1RAP, IL1RN, IL2RA, IL32, IL4I1, IL4R, IL7, IL7R, IL8, IMPA2, IMPACT, INSIG2, IQCG, IQCG, IQGAP1, IQGAP2, IRAK4, IRF7, ISG20, ISLR, ITGA1, ITGA3, ITGA5, ITGAL, ITGB1, ITGB1, ITGB1, ITGB2, ITGB3, ITGB4, ITPKC, ITPRIPL2, ITPRIPL2, JAK3, JUNB, JUNB, KCNE3, KCNE4, KCNMB1, KCNQ1, KCTD10, KDELR1, KDELR2, KDELR2, KHNYN, KHNYN, KIAA0040, KLF10, KLF6, KLF6, KYNU, KYNU, LACTB, LAIR1, LAIR1, LAMA2, LAMB1, LAMB2, LAMC1, LAMP2, LAP3, LAPTM5, LAPTM5, LARP4, LATS2, LATS2, LCP1, LCP2, LDHA, LDHA, LDHA, LEPRE1, LEPREL1, LGALS1, LGALS3, LGALS8, LGALS8, LHFP, LHFPL2, LIF, LILRB1, LILRB2, LILRB3, LIMK2, LIMS1, LIMS3, LITAF, LMAN1, LMAN1, LMNA, LMNA, LMO2, LOC100130633, LOC100131733, LOC100287239, LOC100290115, LOC100292646, LOC100293208, LOC100294102, LOC100294179, LOC100294275, LOC149351, LOC151438, LOC154761, LOC284454, LOC390940, LOC391334, LOC440900, LOC440957, LOC541471, LOC541471, LOC642413, LOC644538, LOC644538, LOC647121, LOC728613, LOX, LOX, LOXL1, LOXL2, LOXL3, LOXL3, LPAR6, LPP, LPP, LRP10, LRRC2, LRRC25, LRRC32, LRRFIP1, LSP1, LTBP1, LTBP2, LTBR, LTF, LUM, LXN, LY75, LY96, LYN, LYZ, M6PR, M6PR, MACC1, MAFF, MAGT1, MAGT1, MAN1A1, MAN1C1, MAN1C1, MAN1C1, MANBA, MAOB, MAP2K3, MAP2K3, MAP3K6, MAP3K6, MAP3K8, MBD2, MBOAT1, MCAM, MCC, MCFD2, MCL1, MDFIC, MDFIC, MEIS3P1, MEIS3P1, MEOX2, METRNL, METTL7B, MFAP3, MFSD1, MFSD7, MGAT4A, MGP, MGST2, MICA, MICALL2, MICALL2, MICB, MICB, MIR155HG, MLKL, MLKL, MMAA, MMP11, MMP14, MMP19, MMP7, MMP9, MNDA, MOBKL1B, MOBKL1B, MOXD1, MPZL1, MPZL2, MPZL3, MR1, MR1, MR1, MRC2, MREG, MS4A14, MS4A4A, MS4A6A, MS4A6A, MS4A7, MSN, MSN, MSN, MSN, MSR1, MSR1, MSR1, MTHFS, MTMR11, MUC1, MVP, MX2, MXRA5, MXRA7, MXRA7, MXRA8, MXRA8, MYADM, MYBPH, MYCBP, MYCBP, MYH9, MYH9, MYL12A, MYL12B, MYL6, MYL6, MYL9, MYO1B, MYO1F, MYO1G, MYO1G, MYOF, MYOF, MYOF, NAAA, NAMPT, NAMPT, NAMPT, NAT1, NCF1, NCF1, NCF2, NCF4, NCRNA00152, NEAT1, NEAT1, NEK6, NEXN, NEXN, NFATC2, NFE2L3, NFE2L3, NFKB1, NFKBIZ, NID2, NLN, NLRC3, NLRC4, NMI, NNMT, NOD1, NOX4, NOX4, NPC2, NPL, NPNT, NQO1, NRP1, NRP1, NRP2, NSUN7, NSUN7, NTAN1, NUAK2, OAS2, OBFC2A, OBFC2A, OCIAD2, OLFML2A, OLFML2B, OLFML3, OPLAH, OSBPL3, OSM, OSMR, OSMR, OSTC, OSTCL, OSTCL, OSTF1, P2RY6, P2RY8, P4HA2, P4HA2, P4HA3, PALLD, PAM, PAMR1, PARP12, PARP14, PARP9, PARVG, PCK2, PCOLCE, PCSK5, PCSK5, PDGFD, PDGFRL, PDIA3, PDIA3, PDIA4, PDIA5, PDK3, PDLIM1, PDLIM4, PDLIM7, PDPN, PDPN, PDZK1IP1, PECAM1, PEX13, PGCP, PGM2, PHF11, PHLDA2, PI4K2B, PIGB, PIGT, PIK3CD, PIM1, PION, PKD2, PLA2G4A, PLA2G5, PLAT, PLAU, PLAUR, PLB1, PLB1, PLBD1, PLEK, PLEK2, PLEKHA4, PLEKHA4, PLEKHA8, PLEKHG1, PLIN2, PLK3, PLOD2, PLP2, PLS3, PLSCR1, PLXDC2, PLXND1, PMEPA1, PNPLA4, PODNL1, PODNL1, PODXL, POM121L8P, POM121L9P, POSTN, PPCS, PPCS, PPIC, PPIC, PPM1M, PPP1CB, PPP1R15A, PPP1R3B, PPP1R3B, PPP2R1B, PQLC3, PRDX6, PRKCDBP, PRKD3, PROCR, PROS1, PROS1, PROS1, PRPS2, PRPS2, PRR16, PRR24, PRSS23, PRSS23, PSD4, PSMB9, PSORS1C1, PTGER4, PTGIR, PTGS1, PTGS1, PTPLAD2, PTPLAD2, PTPN12, PTPN18, PTPN2, PTPN2, PTPN2, PTPN21, PTPN7, PTPRC, PTRF, PTRF, PTX3, PVRL2, PYCARD, PYGL, QTRTD1, RAB11FIP1, RAB22A, RAB27A, RAB32, RAB32, RAB34, RAB36, RAB42, RAB43, RAB43, RAB43, RABGEF1, RABL3, RAC2, RAC2, RAP1B, RAP2C, RARRES1, RARRES2, RASSF5, RBM47, RBMS1, RBMS1, RBP1, RBPMS, RCAN1, RCN3, RDH10, RDH10, RECK, RECQL, REEP5, RELB, RELL1, RER1, REXO2, REXO2, RFTN1, RGS1, RGS16, RGS16, RHOH, RHOJ, RHOJ, RHOQ, RHOQ, RHOQ, RHPN2, RILPL2, RIPK1, RIPK3, RNASE2, RNASE2, RNASE3, RNASE4, RNF135, RNF14, RNF14, RNF144B, RNF149, RNF213, ROD1, ROR1, RPE, RPE, RPL23AP7, RPN1, RPS6KA3, RRAS, RRAS, RRBP1, RREB1, RUNX1, RUNX1, RUNX2, RUNX3, S100A10, S100A11, S100A11, S100A4, S100A6, S100A8, S100A9, SAA1, SAA2, SAMD9, SAMD9, SAMD9, SAMD9L, SAMHD1, SAMSN1, SASH3, SAT1, SAT1, SBNO2, SCIN, SCPEP1, SCYL2, SDC1, SDC2, SDCBP, SEC22A, SEC24D, SEC61G, SECTM1, SEH1L, SERPINA1, SERPINA3, SERPINA3, SERPINA5, SERPINA5, SERPINB1, SERPINB1, SERPINB6, SERPINB6, SERPINB8, SERPINE1, SERPINF1, SERPING1, SERPINH1, SERTAD1, SFRP4, SFT2D2, SGMS2, SH2B3, SH2D4A, SH3GLB1, SHC1, SHISA5, SHOX2, SHROOM3, SIGLEC7, SIGLEC7, SIL1, SIPA1L1, SIPA1L1, SIPA1L2, SKAP2, SLA, SLAIN2, SLAMF8, SLC10A3, SLC11A1, SLC11A1, SLC11A1, SLC12A7, SLC15A3, SLC15A4, SLC15A4, SLC16A10, SLC16A3, SLC16A5, SLC24A6, SLC25A24, SLC25A43, SLC25A43, SLC26A2, SLC2A3, SLC2A9, SLC30A7, SLC35F5, SLC38A6, SLC39A8, SLC41A2, SLC43A3, SLC44A3, SLC46A3, SLC47A2, SLC6A6, SLC7A7, SLFN12, SMC4, SMC5, SMS, SMS, SNAI1, SNAPC1, SNX10, SNX10, SNX20, SNX9, SOAT1, SOCS3, SOD2, SOD3, SP100, SP110, SP140, SP140L, SP140L, SPAG4, SPATA17, SPATS2L, SPCS3, SPHK1, SPINT1, SPOCD1, SPOCD1, SPON2, SPP1, SPPL2A, SPRY1, SQRDL, SRGN, SRPR, SRPX2, SSR3, SSR3, ST14, ST8SIA4, STAC, STAM2, STAT1, STAT3, STAT5A, STEAP1, STEAP3, STEAP3, STK17A, STK38L, STK40, STX3, SUMF1, SUSD2, SVIL, SWAP70, SYK, SYNC, SYNPO, SYTL3, SYTL3, TAGLN, TAGLN, TAGLN2, TAGLN2, TANK, TBC1D1, TBC1D10C, TBC1D19, TBC1D8B, TCEA3, TCEA3, TCEA3, TCIRG1, TCTEX1D1, TDO2, TEAD4, TEP1, TES, TFEC, TFEC, TGFB2, TGFB2, TGFBI, TGFBR2, TGIF1, THBD, THBS1, THBS2, TIFA, TIMP1, TLCD2, TLR1, TLR2, TLR3, TLR6, TLR8, TMBIM1, TMBIM6, TMCO4, TMED5, TMED5, TMED7-TICAM2, TMEM107, TMEM140, TMEM149, TMEM154, TMEM158, TMEM173, TMEM176A, TMEM176B, TMEM2, TMEM220, TMEM220, TMEM43, TMEM49, TMEM64, TMEM67, TMEM70, TMEM71, TMEM87B, TMF1, TMOD3, TMOD3, TMSB10, TMSB4X, TMSL3, TMSL3, TNC, TNFAIP2, TNFAIP3, TNFAIP6, TNFAIP8, TNFRSF10B, TNFRSF10C, TNFRSF11A, TNFRSF11A, TNFRSF11B, TNFRSF11B, TNFRSF12A, TNFRSF14, TNFRSF1A, TNFRSF1A, TNFSF13, TNFSF13B, TNFSF14, TNNI2, TOM1L1, TOM1L1, TPM1, TPM1, TPM1, TPM2, TPM2, TPM3, TPM3, TPM3, TPM3, TPM3, TPM3, TPM4, TPM4, TPM4, TPRG1, TPST1, TRADD, TRAF3IP3, TRAF3IP3, TRAM1, TRAM2, TREM1, TRIM14, TRIM22, TRIM22, TRIM38, TRIM38, TRIM5, TRIM56, TRIM6, TRIM6, TRIP10, TRIP4, TRIP6, TRPC6, TRPM8, TRPV2, TSPAN4, TTC12, TTC12, TTC26, TTC38, TTC39A, TTC39B, TUBA1C, TUBB6, TWISTNB, TWISTNB, TWSG1, TWSG1, TWSG1, TXLNB, TXNDC5, TYMP, TYROBP, UACA, UACA, UBD, UBE2D3, UBE2D3, UBE2F, UBE2H, UBE2H, UBE3C, UGCG, UGCG, UGGT1, UNC93B1, UPP1, UPP1, UTP15, VAMP5, VAMP8, VASN, VASP, VAV3, VAV3, VDR, VEGFA, VEGFA, VEGFA, VIM, VIM, VNN2, VWA1, WARS, WDR1, WDR1, WDR1, WDR66, WDR78, WDR78, WEE1, WIPF1, WIPI1, WTAP, WTAP, WWTR1, WWTR1, XAF1, XAF1, XAF1, XKR8, YIPF1, YKT6, ZAK, ZAK, ZBTB7B, ZC3H12A, ZCCHC9, ZFP36, ZFP36L2, ZNF217, ZNF436, ZNF480, ZNF557, ZNF600, ZNF600, ZNF701, ZNF813, ZNRF2 |
| CGGAseq  n=612 | A2M, ABCD1, SERPING1, C3, CP, CSTB, CYBA, DDB2, DPYD, F13A1, FAH, FTL, FUCA1, GLA, GUSB, HMGCL, SGSH, ICAM1, CFI, IL2RG, ITGB2, JAK3, LYZ, NAGA, NAGLU, SERPINA1, PLOD1, PMM2, PROS1, TGFBI, THBD, WAS, TPP1, CSF2RB, IL2RA, NEU1, PECAM1, C1QB, GALNS, HEXA, HEXB, MAN2B1, SLC11A1, IL10RB, NCF4, SOD2, TGFB1, ANXA1, BLVRB, IL1R1, ITGB7, P4HB, PAM, PLOD2, PPIB, PPIC, PTGER4, NDUFV3, FAM109B, ANXA2, METRNL, PLAUR, APOBEC3F, HCST, GPX8, STEAP3, PIK3R6, CLEC18B, IL32, AGPAT2, LSP1, CARD16, FAM78B, OPA3, MYADM, CHI3L2, TSPAN4, TMEM150A, LIMK2, THEMIS2, CD68, CAST, EIF4G2, MFSD12, PVRL2, TNFRSF1A, TNFRSF1B, PPCS, CASP8, SP100, GPR108, SPI1, DSE, PLOD3, SERPINA3, HRH1, HDAC7, RBM47, EHBP1L1, GGT5, SBNO2, SPATS2L, CXCL16, ACTB, ACTN1, DTX2, IMPDH1, NUDT19, FLNA, TYMP, CTSC, MGAT1, SLC50A1, PPM1M, ADPRH, CFLAR, FLNC, AEBP1, ZNF385A, SLC2A5, FCGR2A, BTBD19, ZBTB42, SLC10A3, MLKL, HSD3B7, PIEZO1, ABCC3, CD58, DOK3, VMO1, EDEM2, TPM4, CSDA, C6orf141, MTMR11, C2, ANXA5, PLEKHA4, SYNPO, CLCF1, OSMR, TCTN1, ARHGDIB, SRPR, RNF135, BAK1, FPR1, ATF5, MR1, ZMYM6NB, SERPINB6, LY96, SIGLEC9, IFI16, CASP4, HLA-A, FAM20A, FHL3, HLA-DRB1, TNFSF8, MAP3K8, CD40, CREB3L2, IGFBP7, CD300A, VSIG4, EPS15L1, PSMD9, NTAN1, JAKMIP2, FCGR3B, DENND2D, CHI3L1, CLIC1, CCR1, CTSZ, DPAGT1, EMP1, EMP3, B4GALT1, HSPB1, CYR61, IL13RA1, ACP2, AIM1, ARF6, CFB, BGN, C1R, C1S, C5AR1, CD72, CDH11, CD52, CTSB, CTSD, ETV6, FPR3, GBP1, GCLM, GNA15, GNS, GRN, HK3, HLA-DMB, HLA-DOA, HLA-DPB1, ICAM3, IL7R, IRF1, ISG20, ITGA5, JUNB, LAIR1, LAMB2, LAMC1, LCP1, LGALS1, LOX, LOXL2, MMP19, MSN, MSR1, MYH9, SLC22A18, FURIN, PLAU, PLP2, SRGN, MAPK13, PTPN9, PYGL, RAC2, RAP2B, RBMS1, RGS2, RNASE2, RNASE4, RPN1, S100A4, S100A10, SAT1, SECTM1, SPAG4, SRF, TRIM21, STAT3, STAT5A, TEAD3, THBS1, TLR1, TLR2, TRIP6, UGCG, VASP, ZFP36, ADAM12, TPST1, GPR65, CST7, BHLHE40, KMO, RNASET2, SOCS1, VAMP8, STX11, TRADD, RIPK1, TNFSF14, TNFRSF14, SIGLEC5, IQGAP1, FCGBP, BCL10, MBD2, STBD1, ATP6V0E1, MAP3K14, SOCS3, DOK2, B2M, PLK3, FCER1G, GBP2, PSMB8, TRIP10, CD163, CHST2, CYTIP, CALR, CTBS, DAP, DUSP1, GALNT2, RARRES3, MAP3K6, NMI, PCSK7, MAPKAPK2, ARHGAP29, KCNK6, GMFG, PDIA4, ACTN4, MMP14, PFN1, PIK3CD, WDR1, MVP, CLEC2B, BCL3, CEBPD, CSTA, DDOST, ELK3, FOSL2, GNG5, PDIA3, HCLS1, RAB8A, MAFB, SH2B3, TOM1L1, HLA-B, HLA-E, IFNGR2, LCP2, LGALS3BP, RNASE6, S100A11, ST8SIA4, ARPC1B, NAMPT, LHFPL2, FLOT1, FMNL1, MANBA, MICB, SNAI1, MANF, TCIRG1, NOD1, HLA-DMA, CAPZA1, DNAJB1, PRICKLE3, NNMT, NUCB1, DNAJC3, RRAS, TLN1, TNFAIP2, FBLN5, IFI30, TRIM38, BATF, PROCR, NPC2, IFITM2, MYL12A, RELB, ZNF217, CD226, SLC12A7, VAMP5, CD300C, LAPTM5, MARCO, KDELR1, PDIA5, LMAN2, CKAP4, RAB32, GLIPR1, RIPK3, ZFP36L2, C10orf10, TMEM115, FSTL1, SSR3, PRSS23, TWF2, CD93, BACE2, CD2AP, PTRF, PLA2G15, MYO1F, PLXNB2, ICMT, FHOD1, CLEC5A, ZDHHC1, SEC61A1, SERTAD1, CYTH4, MYOF, LRP10, ORMDL2, CD5, TRAM1, ETHE1, HEBP2, PPP1R15A, SIGLEC7, CIDEB, APOBEC3C, TMOD3, LATS2, EHD2, EDEM1, ACAP1, KIAA0247, SEC24D, CHSY1, DENND3, PLXND1, 38961, STAB1, RFTN1, NBEAL2, MAN2B2, DPY19L1, KHNYN, OLFML2B, ATL3, REXO2, CCDC9, TES, GADD45B, LAP3, CALHM2, C1QA, TRPV2, CPQ, SDF4, SNX9, CPA4, COPZ2, PLEK2, C1RL, SLC15A3, TNFRSF12A, ZAK, EFEMP2, F11R, PION, TMED9, OPLAH, QPCTL, TOR4A, CMTM6, RAB20, TTC12, SLC35F6, CCDC109B, TTC38, WIPI1, TMEM248, SLFN12, XKR8, SHQ1, DRAM1, ADAP2, ARHGAP15, SLC16A10, APOBR, SASH3, CHPF2, ADAMTSL4, HLA-DRA, TMEM234, SLAMF8, RNPEP, FAM20C, GPR84, MAN1C1, IFITM3, PLSCR1, TMSB4X, SQRDL, TMEM8A, SCPEP1, APOBEC3G, FKBP10, HLX, ST14, DEF6, NXF3, TMBIM1, EPS8L2, VKORC1, YIPF2, TMEM109, TMEM43, LRRC2, FYCO1, MUL1, ACSS3, GAL3ST4, GLT25D1, IGFLR1, PLBD1, RIN3, DENND1C, DNAJC22, SLC24A6, FAM214B, ITPKC, ULBP2, REEP4, PDCD1LG2, CYP2S1, SLC2A10, UNC93B1, TRIM56, HDHD3, ADPGK, SH3BGRL3, FERMT3, GRWD1, STK40, FUCA2, MFSD7, ZDHHC18, CARD6, LOXL3, TUBA1C, HAVCR2, PARP10, ZDHHC12, SMIM3, SYDE1, MYO1G, CCDC102A, PML, TNKS1BP1, FCHSD1, ARHGAP18, ATG4A, FAM46B, FAM129A, GIT2, KCNE4, MOB3A, DTX3L, FAM114A1, SHKBP1, SP140L, CMTM7, KLHDC7B, VASN, ARL11, GALM, EHD4, CMTM3, SLFN5, TMEM106A, RILPL2, CABP4, LRRC25, SLC25A43, NFAM1, GJD3, PQLC3, HGSNAT, APOBEC3D, GAPT, CNPY4, IKBIP, RDH10, RNF149, CRB2, CD300LB, C1orf162, TXLNA, CCDC125, TMCO4, TMEM255B, RNF144B, TICAM1, MICALL2, TMEM37, CLDN23, ABHD15, TMEM173, EMB, ZNF600, TPRG1, CA13, LRRN4CL, YY2, ARID5A, CMAHP, NCF1C, LOC154761, LBX2-AS1, C21orf88, C1RL-AS1, TREML3P, NEAT1, MIR22HG, CD99P1, LOC284751, LOC283143, LOC100507463, LOC100506385, LOC100506585, LOC100505812, LOC100130476, SAA1 |
