# Supplementary material for: Immunological and clinicopathological characteristics of C1RL in 2120 glioma patients
Source: BMC Cancer. 2020 Sep 29;20:931. doi: 10.1186/s12885-020-07436-6 (PMC7526369; doi:10.1186/s12885-020-07436-6)
Supplement: Supplementary file 3 — Additional file 3 Table S3. C1RL and WHO grade and glioma histopathology. [file 12885_2020_7436_MOESM3_ESM.docx]

S Table 3. C1RL and WHO grade and glioma histopathology

|  |  | TCGAseq n=663 | | | CGGAmic n=259 | | | CGGAseq n=219 | | |
| --- | --- | --- | --- | --- | --- | --- | --- | --- | --- | --- |
|  |  | Low | High | P value | Low | High | P value | Low | High | P value |
| Grade | 2 | Unkown | Unkown | Unkown | 89 | 27 | P<0.0001 | 70 | 19 | P<0.0001 |
|  | 3 | Unkown | Unkown |  | 21 | 14 |  | 27 | 20 |  |
|  | 4 | Unkown | Unkown |  | 20 | 88 |  | 13 | 70 |  |
| histopathology | O and AO | 145 | 43 | P<0.0001 | 19 | 8 | P<0.0001 | 30 | 3 | P<0.0001 |
|  | OA and AOA | 86 | 44 |  | 38 | 17 |  | 42 | 18 |  |
|  | A and AA | 94 | 99 |  | 53 | 16 |  | 25 | 18 |  |
|  | GBM | 7 | 145 |  | 20 | 88 |  | 13 | 70 |  |

The differences were compared with Chi-square test.
